# Location and timing govern tripartite interactions of fungal phytopathogens and host in the stem canker species complex

**DOI:** 10.1186/s12915-023-01726-8

**Published:** 2023-11-07

**Authors:** Elise J. Gay, Noémie Jacques, Nicolas Lapalu, Corinne Cruaud, Valerie Laval, Marie-Hélène Balesdent, Thierry Rouxel

**Affiliations:** 1 Université Paris-Saclay, INRAE, UR BIOGER, 91120 Palaiseau, France; 2 Genoscope, Institut François Jacob, CEA, Université Paris-Saclay, Evry, France

**Keywords:** *Leptosphaeria maculans*, *Leptosphaeria biglobosa*, *Brassica napus*, Fungal biology, Transcriptomics, Tripartite interactions, Competition, Pathogenicity program, Plant defense responses

## Abstract

**Background:**

*Leptosphaeria maculans* “brassicae” (Lmb) and *Leptosphaeria biglobosa* “brassicae” (Lbb) make up a species complex involved in the stem canker (blackleg) disease of rapeseed (*Brassica napus*). They coinfect rapeseed together, from the early stage of infection on leaves to the final necrotic stage at the stem base, and both perform sexual crossings on plant residues. *L. biglobosa* is suggested to be a potential biocontrol agent against Lmb, but there has been no mechanistic investigation of the different types of interactions that may occur between the plant and the two fungal species.

**Results:**

We investigated the bi- or tripartite interaction mechanisms by (i) confronting Lmb and Lbb in culture conditions or during cotyledon infection, with different timing and/or spore concentration regimes, (ii) performing RNA-Seq experiments in vitro or on the kinetics of infection of cotyledons infected by Lmb and/or Lbb to evaluate the transcriptomic activity and the plant response when both fungal species are inoculated together. Lbb infection of *B. napus* cotyledons was typical of a necrotrophic behavior, with a very early setup of one pathogenicity program and very limited colonization of tissues. This contrasted with the complex succession of pathogenicity programs of the hemibiotroph Lmb. During simultaneous co-infection by both species, Lmb was strongly impacted in its growth and transcriptomic dynamics both in vitro and *in planta*, while Lbb was unaffected by the presence of Lmb. However, the drastic inhibition of Lmb growth by Lbb was ineffective in the case of delayed inoculation with Lbb or a lower amount of spores of Lbb compared to Lmb.

**Conclusions:**

Our data suggest that Lmb growth inhibition by Lbb is the result of a combination of factors that may include competition for trophic resources, the generation by Lbb of an environment unsuitable for the lifecycle of Lmb or/and the effect on Lmb of plant defense responses induced by Lbb. It indicates that growth inhibition occurs in very specific conditions (i.e., co-inoculation at the same place of an equal amount of inoculum) that are unlikely to occur in the field where their coexistence does not prevent any species from completing their life cycle.

**Supplementary Information:**

The online version contains supplementary material available at 10.1186/s12915-023-01726-8.

## Background

Arable crops are subjected to numerous fungal diseases usually studied as a bi-partite interaction between a fungal pathogen and its host. However, plants are usually co-infected by multiple pathogens simultaneously in different tissues or stages of plant growth. While, in biology, species complexes usually refer to a group of similar organisms, that may be difficult to resolve in terms of species, phytopathology often uses “species complexes” for closely related pathogen species co-infecting a plant species [1, 2]. Species complexes are extensively studied in bacteria or viruses [2], and only a few fungal diseases are thoroughly investigated. Yet, several species complex of fungal pathogens cause major plant diseases, so that it is sometimes difficult to disentangle the contribution of each species to symptom development and subsequent damage to the crop. For instance, a species complex involving *Mycosphaerella pinodes* and *Phoma medicaginis* var. *pinodella* causes the Ascochyta blight disease in peas [3]*, Oculimacula yallundae* and *O. acuformis* are involved in the eyespot disease of wheat [4], the *Erysiphe* pathogenic complex causes powdery mildew of oak [5], and *Fusarium graminearum. F. pseudograminearum*, *F. culmorum*, *F. poae,* and *Microdochium nivale* are involved in the Fusarium head blight of wheat [6].

The intimate relationship among species of species complexes suggests that fungus-fungus interactions or more complex multi-partite fungus-host interactions could impact symptom expression and the ultimate disease [2]. Interactions between pathogens can take various forms, such as synergism [7], global lowering of virulence, or direct/indirect promotion of one species over another [2, 8]. For example, molecular screenings on natural populations revealed various interaction types between species involved in the *Fusarium* head blight, including synergism or indirect competition, as shown by the significant co-occurrence of *F. graminearum* and *F. culmorum* in Poland [9] or the opposite occurrence between *F. graminearum* and *F. poae* under a heat/humidity gradient [10]. Controlled inoculations, such as concomitant or sequential co-infections on local or distant tissues in varying abiotic conditions, are also used to decipher the mode of interaction and identify the effect on plant damages and pathogen reproduction. For example, disease development varied if *M. pinodes* and *P. medicaginis* var. *pinodella* were inoculated together or sequentially on pea [3]. Indirect interactions through the plant responses were also demonstrated in several cases of infection by multiple species, revealing that plant responses can promote or inhibit one species over another. Thus, some species activate defense pathways that limit new co-infections, as observed for many beneficial organisms, especially symbiotic organisms [11].

**Fig. 1.**
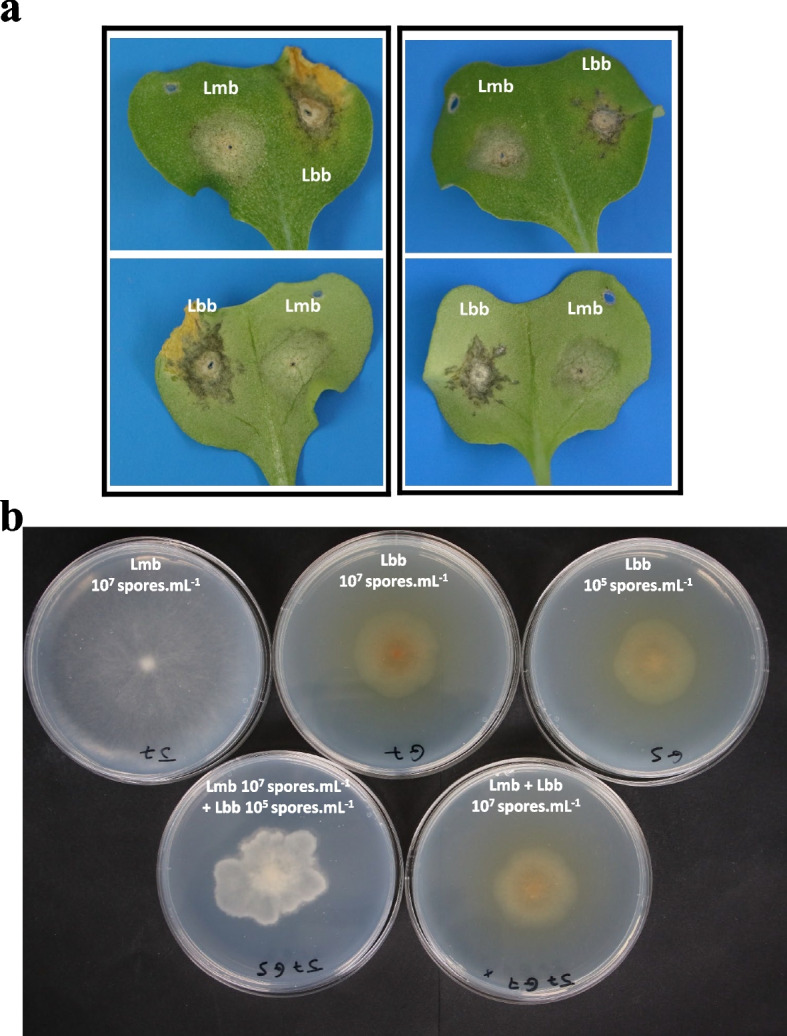
Symptoms on cotyledons and morphology in vitro of *Leptosphaeria maculans* “brassicae” (Lmb) or/and *Leptosphaeria biglobosa* “brassicae” (Lbb). **a** Examples of symptoms caused by current French field isolates of Lmb and Lbb on cotyledons of two winter-type genotypes (left, line 15–23-4–1 and right, line 18–22-6–1). Pictures were taken 9 days after inoculation and lower panel pictures are reverse face of the upper ones. **b** Colony morphology of Lmb, Lbb, and mixes of the two species. The isolates were deposited as droplets of 10^7^ spores.mL^−1^ pycnidiospore suspension, as 10^5^ spores.mL^−1^ pycnidiospore suspension or as a mix of 10^7^ spores.mL^−1^ Lmb pycnidiospore suspension + 10^5^ spores mL^−1^ Lbb pycnidiospore suspension, and grown for 14 days on agar MMII medium

The *Leptosphaeria* species complex causing stem canker (aka. blackleg) disease on *Brassica napus* encompasses species with contrasting lifestyles. *L. maculans* “brassicae” (Lmb) and *L. biglobosa* “brassicae” (Lbb) are systematically found associated on *B. napus* crops in Europe [12], while the association between Lmb and other *L. biglobosa* subspecies such as *L. biglobosa* “canadensis” (Lbc), can be found on other continents [13]. In Europe, both species coexist on their host all along the oilseed rape life cycle, from the seedling stage in autumn, where the primary leaf infection takes place (Fig. 1a), to the adult stage in spring, when the stem canker develops [12, 14]. During the early infection stage on leaves, Lmb has a hemibiotrophic lifestyle, whereas Lbb is considered a strict necrotroph, and typical symptoms are gray-green collapses for Lmb and dark necrotic leaf spots for Lbb (Fig. 1a). Then during several months (from autumn to spring), both fungi have been shown (for Lmb) or hypothesized (for Lbb) to migrate from the leaves through the petiole to the stems with an endophytic/asymptomatic behavior [15–17]. At the end of spring, they both display a necrotrophic lifestyle and cause necrotic lesions, described in the literature as either basal stem canker for Lmb or upper stem lesions for Lbb [14]. However, Lbb is present in the basal stem tissues at the end of the growing season, along with Lmb, and its contribution to basal stem canker (if any) is somehow obscure at present [12, 18].

Several studies based on symptom quantification in controlled conditions or *in natura* hypothesized that Lbb and Lbc compete with Lmb and limit its incidence [19, 20]. In these studies, co-infection tests under controlled conditions suggested the occurrence of indirect, plant-mediated interactions between the two species: pre-inoculations on the same or on different leaves by Lbb or Lbc significantly reduced the size of lesions caused by Lmb [19–22]. In contrast, analyses of agricultural practice in France, using fungal biomass quantifications, did not confirm the negative interaction between the two species on leaf or stem samples over 2 years of monitoring [12]. Such studies, however, are faced with great variability in the proportion or occurrence of the different members of the complex in time (within and between growing seasons [12]) or in space (regional variations [23, 24]), making it more difficult to conclude on the type of interaction between members of the complex. Very few competition studies between Lmb and *L. biglobosa* have been performed in vitro. Antagonism was found between Lmb and fungal species such as *Alternaria brassicicola* or *L. biglobosa*, and 13-day-old cultures of Lmb were found to inhibit the germination of *L. biglobosa* “australensis” [25]. According to the authors, this was attributable to the production of a toxin, sirodesmin PL (and possibly other secondary metabolites), by Lmb [25].

If actual antagonistic interactions occur, the parameters underlying the reduction of symptoms of Lmb during co-infection, and whether Lbb interferes with Lmb pathogenesis via toxinogenesis, are still unknown to date [25]. Transcriptomic approaches allow dissecting gene expression in samples containing several organisms simultaneously. It also provides a global view of gene expression changes when the molecular determinants of the observed interaction are unknown. For instance, it has been shown that the activation of genes encoding proteins involved in plant defense responses is probably involved in the antagonistic interaction observed between *Macrophomina phaseolina* and an arbuscular mycorrhizal fungus [26]. Also, the co-infection of two fungal antagonists, *Pseudozyma flocculosa* and *Blumeria graminis*, on barley was analyzed by transcriptomics and revealed a complex tripartite interaction. From these data, the authors were able to develop an interaction model involving the three protagonists: *P. flocculosa* effectors have a deleterious effect on the development of *B. graminis* haustorium, which deregulates the plant’s response to the benefit of *P. flocculosa* [27].

Recently, we undertook a large-scale transcriptomic analysis to dissect the whole life cycle of Lmb in interaction with its host plant [28]. This study allowed a complete and precise dissection of all stages of the Lmb life cycle [28]. In contrast, very few similar data are available for Lbb, with only one transcriptomic analysis at two dates of cotyledon infection by Lbc, confirming the necrotrophic lifestyle of Lbc [29]. In addition, plant response to Lmb and Lbc species individually displayed the typical response to hemibiotrophic and necrotrophic fungi, respectively [29]. Available genomic and transcriptomic data have provided information on the two fungal species individually, but, to date, no molecular information is available to determine the mechanisms underlying the competitive interaction previously hypothesized between both species.

In this paper, we investigate the mechanisms of the bi- or tripartite interaction by (i) confronting Lmb and Lbb in culture conditions or during cotyledon infection, with different timing or spore concentration regimes; on these, we performed quantifications of fungal biomasses, macro-, and microscopic observations using GFP- and RFP- isolates. We also performed RNA-Seq experiments on three kinetics of infection of cotyledons infected by Lmb and/or Lbb to (ii) refine expression patterns of Lbb and *B. napus* during cotyledon infection and (iii) evaluate the transcriptomic activity and the plant response when both fungal species are inoculated together. Our data strongly suggest that the inhibition of Lmb growth observed when both species are present, either in vitro or in planta, involves a combination of factors. They also suggest that growth inhibition only occurs in very specific conditions, i.e., co-inoculation at the same place with an equal amount of inoculum, which is very unlikely to occur in field conditions.

## Results

### In vitro biological interactions between *L. maculans* ‘brassicae’ (Lmb) and *L. biglobosa* ‘brassicae’ (Lbb)

During in vitro SSI, growth rates were similar between Lmb and Lbb on the V8 medium, while Lmb had a faster radial growth compared to Lbb on the MMII medium (Additional file 1: Fig. 1; Fig. S1a). In a condition of equal concentration of spores (eMSI), Lbb growth overran the development of Lmb, for which GFP-spores and hyphae were restricted to the inoculation point (Additional file 2: Fig. S2). Lbb mycelia colonized the Petri dish with the same kinetics in eMSI and SSI conditions (Additional files 1 and 2: Figs. S1 and S2). When lowering the Lbb spore concentration (uMSI), Lbb and Lmb mycelia grew at a rate similar to that of Lbb SSI but did not produce the typical Lbb pigment, and the colonies often had uneven perimeter (Fig. 1d). In addition, RFP-Lbb and GFP-Lmb mycelia intermingled without showing signs of exclusion (Additional file 2: Fig. S2). Finally, during delayed co-inoculation (dMSI), only a dense network of GFP-Lmb hyphae was visible (data not shown).

In confrontation experiments, there was no growth inhibition of Lmb by Lbb, but an exclusion front between the two species was evident, and the hyphae from both species did not merge (Additional file 3: Fig. S3). This was confirmed with two other Lbb isolates (ATI-19–322 and INV-17–199; data not shown). Lastly, no effect could be detected on the pycnidiospore germination rate of each species by the other in vitro (data not shown).

RNA-Seq data obtained in vitro in SSI and MSI showed that, at 7 days of culture, an average of 99.35% of raw reads were assigned to Lbb in MSI (Additional file 4: Table S1), and no genes were over or under-expressed in the presence or absence of Lmb in these in vitro conditions.

### *In planta* biological interactions between *L. maculans* and *L. biglobosa*

**Fig. 2.**
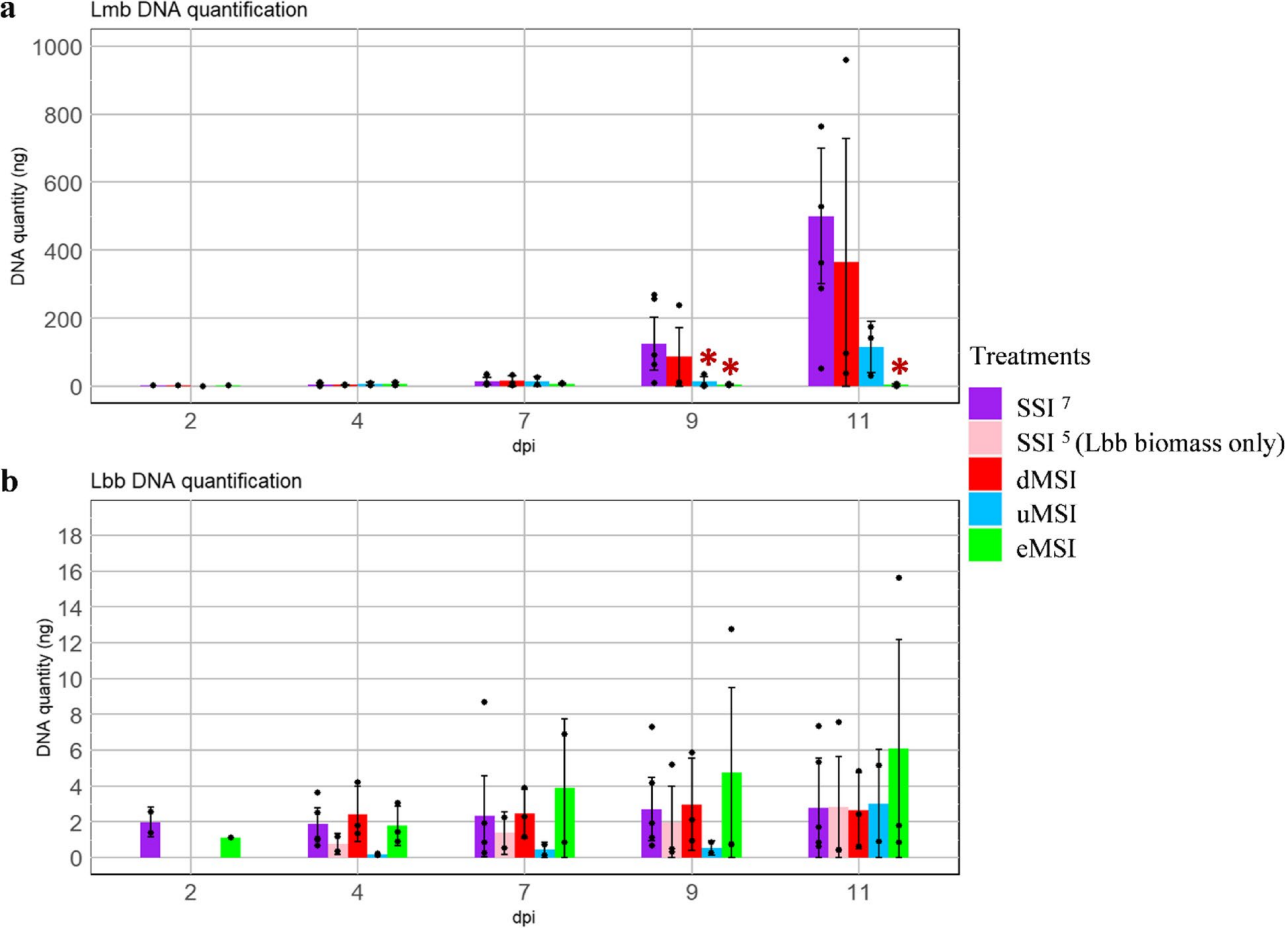
Biomass quantification of *Leptosphaeria maculans* “brassicae” and *Leptosphaeria biglobosa* “brassicae” during colonization of *Brassica napus*. Quantifications of DNA of *Leptosphaeria maculans* “brassicae” (Lmb) (**a**) and *Leptosphaeria biglobosa* “brassicae” (Lbb) (**b**) were done at 2, 4, 7, 9, and 11 days post-inoculation (dpi) of *Brassica napus* cv. Darmor-*bzh*. Single-species inoculation (SSI) were done by inoculating pycnidiospore suspensions of Lmb or Lbb at 10^7^ pycnidiospores.mL^−1^ (SSI, purple bars) or at 10^5^ pycnidiospores.mL^−1^ (SSI, pink bars, not done for Lmb). Mixed species inoculations (MSI) were as follows: (i) delayed MSI with an addition of Lbb inoculum 2 days after the inoculation of Lmb (dMSI, red bars); (ii) concomitant MSI with different spore concentrations: Lmb at 10^7^ pycnidiospores.mL^−1^ and Lbb at 10^5^ pycnidiospores.mL^−1^ (uMSI, blue bars); (iii) concomitant MSI with equal concentration of pycnidiospores (mix of 10^7^ pycnidiospores.mL^−1^ for each species) (eMSI, green bars). DNA quantities were measured by qPCR using partial sequence of the *EF1α *gene for Lmb, and of the actin gene for Lbb, using six biological replicates for SSI conditions and three biological replicates for the three MSI conditions. Each black dot corresponds to individual values for each replicate in a given condition. Pairwise statistical comparisons were done on the average DNA quantities obtained on all replicates in each condition and at each timepoint. Statistical analyses were done with the non-parametrical Wilcoxon test. The asterisks represent a significant difference compared to the control SSI (*p* < 0.05). The error bars represent the standard deviation

Following Lmb SSI, symptoms began to be visible 9 days post-inoculation (dpi), and the biotrophic/asymptomatic stage (4–9 dpi) was easily distinguishable from the necrotrophic stage (9–14 dpi) (Additional file 5: Fig. S4a). At the penetration stage, (2 and 4 dpi) GFP-expressing pycnidiospores aggregated and numerous germ tubes developed in the intercellular space of epidermal cells (Additional file 5: Fig. S4a). Subsequently, hyphae grew between plant cells from 7 to 9 dpi and massively colonized the intercellular spaces. At this stage, typical leaf spots developed, with pycnidia differentiation (Additional file 5: Fig. S4a). Lmb biomass in cotyledons increased slightly between 2 and 7 dpi and then increased drastically between 9 and 11 dpi, reaching an average amount of 500 ng at 11 dpi (Fig. 2; Additional file 6: Table S2).

For Lbb during SSI, black necrotic patchy symptoms, contrasting with the grayish symptoms caused by Lmb, developed much earlier than those of Lmb (4 and mostly 7 dpi) and were accompanied by a chlorotic halo of variable size absent for Lmb SSI (Additional file 5: Fig. S4b). Lbb biomass was very low within plant tissues and did not significantly increase with time despite the large macroscopic symptoms (Fig. 2b; Additional file 6: Table S2). In our conditions, no or very low amounts of Lbb pycnidia differentiated.

In the case of eMSI, symptoms appeared at 4, and mostly 7 dpi with the same kinetics and phenotype as those observed for Lbb SSI (Additional file 5: Fig. S4c). Lmb pycnidiospores from the primary inoculum were visible whatever the time point. In some plants, Lmb hyphae were visible starting from 9 dpi, but they remained confined to the surroundings of the inoculation point. Finally, no differentiation of Lmb pycnidia was observed on leaf spots (Additional file 5: Fig. S4c). Co-inoculation of Lmb and Lbb had a drastic impact on Lmb biomass compared to Lmb SSI, with no significant increase between 2 and 11 dpi, while it had no measurable impact on Lbb biomass (Fig. 2; Additional file 6: Table S2).

In the case of uMSI, no differences in Lmb mycelium and conidia development could be visually detected compared to the control Lmb SSI (Additional file 5: Fig. S4ad). Lmb DNA amounts increased more slowly than in the SSI control, but the differences were not significant at *p* < 0.05 (Fig. 2; Additional file 6: Table S2). Dark patches, characteristic of Lbb lesions, were observed following uMSI, suggesting that the overrepresentation of Lmb spores did not inhibit the Lbb development (Fig. 2).

When delayed inoculations were done by inoculating Lbb 2 days after Lmb (dMSI), symptoms appeared 7 days after Lmb inoculation. We could discriminate small necrotic lesions caused by Lbb around the inoculation spot, suggesting that Lbb could infect cotyledons but in constrained areas (Additional file 5: Fig. 4e). However, Lmb was not impacted by the Lbb development and pursued the cotyledon infection similarly to Lmb SSI infection. Consistent with these results, the delayed co-inoculation with Lbb did not induce significant differences in DNA quantities compared to the control SSI, either for Lbb or Lmb (Fig. 2).

### Quantitative aspects of the plant infection as revealed by RNA-Seq

The proportions of fungal reads assigned to Lmb or Lbb following SSI on cotyledons were very low at 2 and 5 dpi for Lmb (Additional file 7: Fig. S5a) and until 7 dpi for Lbb (Additional file 7: Fig. S5b). It then increased steadily to reach 80.9 to 82.6% of the reads at 15 dpi for Lmb (Additional file 7: Fig. S5a). An increase was also observed for Lbb reads with time, beginning at 9 dpi or 12 dpi, depending on the replicate, suggesting a similar but delayed increase in one replicate compared to the other (Additional file 7: Fig. S5b). To ensure that the variability between replicates in Lbb read count did not generate biases for further analysis, we performed a linear regression, which showed that the pairwise correlation of replicates was no lower than 0.90 (Additional files 8 and 9: Table S3, Text S1).

Following eMSI, the proportion of fungal (Lmb + Lbb) reads in the samples followed a trend similar to that observed following Lbb SSI, including variability between replicates, but delayed in timing. In addition, the proportion of total fungal reads in the samples was lower than what was observed following Lbb or Lmb SSI (Additional files 4 and 7: Table S1, Fig. S5c). In addition, the proportion of Lmb reads over Lbb reads steadily decreased with time during the co-infection, departing from 46.2% for Lmb and 53.8% for Lbb at 2 dpi and reaching 12.3% for Lmb and 87.7% for Lbb at 15 dpi (Additional files 4 and 7: Table S1, Fig. S5d). Compared to what was obtained during eMSI in vitro, with less than 1.22% of the reads assigned to Lmb 7 dpi, 27% of the fungal reads were assigned to Lmb 7 dpi following eMSI on cotyledons (Additional file 4: Table S1).

### *B. napus* responses to infection by Lbb or Lmb

**Fig. 3.**
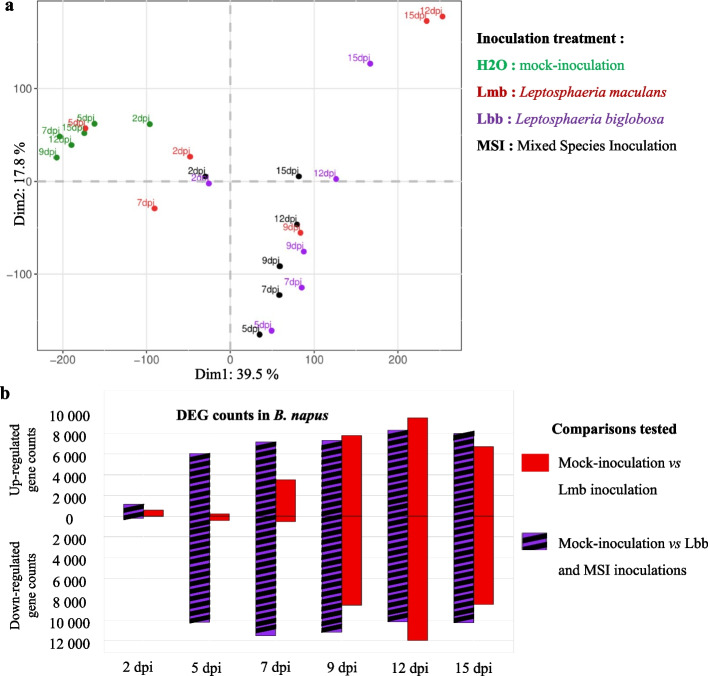
Analysis of *Brassica napus* gene expression following inoculation by *Leptosphaeria maculans* or/and *Leptosphaeria biglobosa*. RNA samples were collected at 2, 4, 7, 9, 11, and 15 days post-inoculation (dpi) following inoculation of *Brassica napus* cv. Darmor-*bzh* cotyledons. Single-species inoculations (SSI) were done by inoculating pycnidiospore suspensions of *Leptosphaeria maculans* “brassicae” (Lmb) or *Leptosphaeria biglobosa* “brassicae” (Lbb) at 10^7^ pycnidiospores.mL^−1^. Mixed species inoculation (MSI) were done by mixing an equal volume of Lmb and Lbb spore suspensions at 10^7^ pycnidiospores.mL^−1^. **a** Principal component analyses (PCA) were done on normalized gene expression of *B. napus* (centered Log_2_(FPKM + 1)). The color code is as follows: SSI by Lbb (purple), SSI by Lmb (red), MSI (black), and control mock inoculation with water (green). **b** For each of the six time points, the resulting number of differentially expressed genes (DEGs) (*p* < 0.05, LogFC >|2|) is shown by the histogram which includes the DEG set detected between control samples and Lmb SSI in red and the DEG set detected between the control samples and the “Lbb SSI + MSI” samples in hatched black and purple bars.

PCA (Fig. 3a) and detections of differentially expressed genes (DEGs) (Fig. 3b) provided a first information about the plant response to each species. The PCA firstly showed that, at 2 dpi, control samples grouped with inoculated ones (SSIs or MSI), and no DEGs were detected between any pairs of treatments, suggesting there is no sensing by the plant of the infections at this very early stage. At all time points, control samples grouped together suggesting that there were no intrinsic transcriptomic changes with time when cotyledons were inoculated with water (Fig. 3a). At 5 dpi, *B. napus* samples from Lmb SSI were also close to control samples, and a low number of DEGs were detected (Fig. 3b). The response of *B. napus* to Lmb SSI was most evident at 7 dpi with 4051 DEGs and reached a peak at 12 and 15 dpi with 21,467 and 15,212 DEGs detected, respectively (Fig. 3b). In contrast, plant DEGs were detected starting at 5 dpi in response to Lbb SSI (16,265 DEGs) and lasted until the end of the kinetics (Fig. 3a,b). All MSI and Lbb SSI samples were grouped in the PCA (Fig. 3a), and no DEGs were detected between these two conditions, whatever the time point.

### Common *B. napus* responses to inoculation by *L. maculans *or *L. biglobosa*

**Fig. 4.**
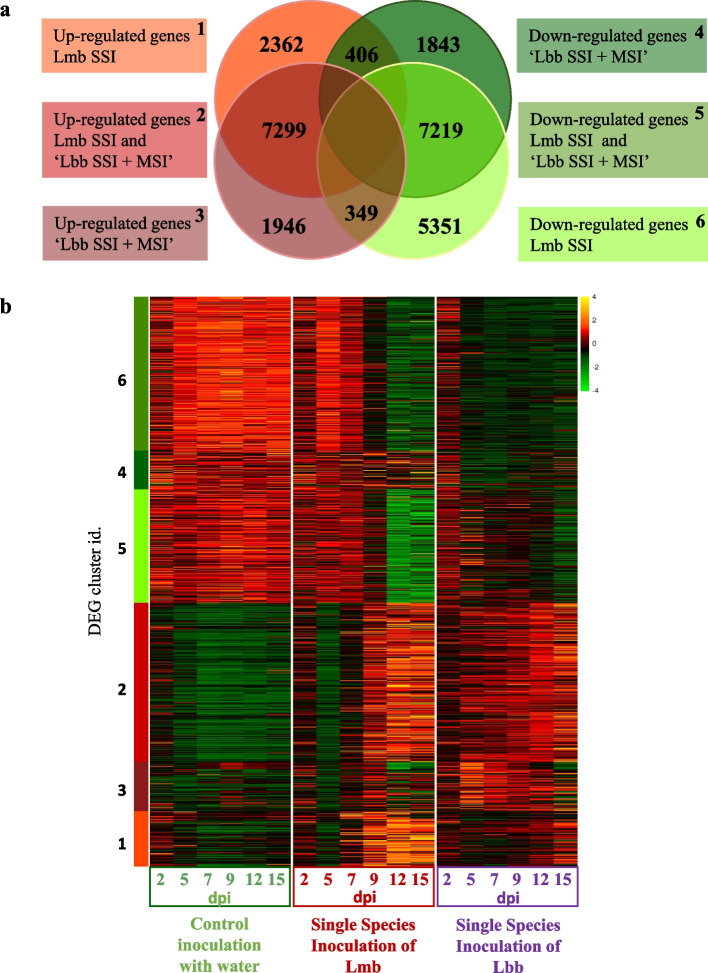
Heatmap of *Brassica napus* genes differentially expressed following inoculation by *Leptosphaeria maculans* and *Leptosphaeria biglobosa*. RNA samples were collected at 2, 4, 7, 9, 11, and 15 days post-inoculation (dpi) following inoculation of *Brassica napus* cv. Darmor-*bzh* cotyledons. Single-species inoculations (SSI) were done by inoculating pycnidiospore suspensions of *Leptosphaeria maculans* “brassicae” (Lmb) or *Leptosphaeria biglobosa* “brassicae” (Lbb) at 10^7^ pycnidiospores.mL^−1^ and mixed species inoculations (MSI) were done by mixing an equal volume of Lmb and Lbb spore suspensions at 10^7^ pycnidiospores.mL^−1^. **a** The total number of differentially expressed genes (DEGs) was obtained according to two statistical comparisons: for each of the six sampled timepoints, (i) gene expression in cotyledons inoculated with Lbb (SSI and the MSI) was compared to that of the control cotyledons inoculated with water and (ii) gene expression in cotyledons inoculated by Lmb was compared to that of control cotyledons inoculated with water. From these differentially expressed gene sets, the Venn diagram illustrates the intersection of the up- or downregulated genes in response to Lmb SSI (1,6); in response to “Lbb SSI + MSI” (3, 4), and in response to both Lmb SSI and “Lbb SSI + MSI” (2,5). **b** The heatmap represents the normalized expression (centered Log_2_(FPKM + 1)) of DEGs in *B. napus* following mock inoculation with water (left block), and during SSI by Lmb or Lbb (middle and right blocks, respectively). Genes were grouped in the six categories highlighted by the Venn diagram, and identified by the bar on the left of the heatmap with the same color code as in the Venn diagram

The expression waves of DEGs in *B. napus* in response to the kinetics of infection by Lmb SSI or by both Lbb SSI—MSI compared to control condition first highlighted a large set of common up- or downregulated genes (14,518 common DEGs, 54% of total DEGs) (Fig. 4a). However, this common DEG set displayed specific expression profiles depending on the infection treatments (Fig. 4b).

The 7299 genes over-expressed in common in response to the three infection conditions were mobilized very early (5 dpi) in response to Lbb, while they were strongly induced only at 9 dpi following Lmb SSI (Fig. 4b). This gene set was strongly enriched in genes involved in various defense responses (response to a drug, to abiotic stimuli, to stress, to organisms such as bacterium or fungus, all with a corrected *p* < 1.e^−40^, Additional file 10: Fig. S6). In addition, among the GO terms belonging to Molecular Processes, a strong enrichment was detected in Serine Threonine Kinase activity (*p* = 1.e^−9^, Additional file 11: Fig. S7), a large family known to be involved in the detection and signaling transduction of defense responses following PAMP (pathogen-associated molecular pattern) recognition [30].

Similarly, downregulation was only observed departing from 9 dpi for Lmb SSI, while it was evident departing from 5 dpi following Lbb SSI (Fig. 4b). This set of 7219 genes was enriched in primary carbon metabolism processes (photosynthesis, response to light, generation of precursor metabolites and energy, photosynthetic electron transport chain; all with a corrected *p* < 1.e^−30^; Additional files 12 and 13: Figs. S8 and S9).

### Specific *B. napus* responses to inoculation by *L. maculans* or *L. biglobosa*

Genes involved in the specific responses to Lmb or “Lbb and MSI” (between 1843 and 5351 DEGs, Fig. 4a) harbored the same expression profiles (on/off during the fungal switch to necrotrophy), and similar GO term enrichments as in the common responses (Additional files 10, 11, 12 and 13: Figs. S6, S7, S8, and S9). Although two unique sets of genes can indeed be distinguished in response to Lmb or “Lbb and MSI,” the GO enrichment analysis could not highlight specific metabolic or defense pathways associated with these two sets of genes (Additional files 10, 11, 12, 13 and 14: Figs. S6, S7, S8 and S9, Text S2). The main specific response towards Lmb compared to Lbb regarded the downregulation of numerous genes involved in responses to hormonal signalization (response to endogenous stimuli, response to hormones, hormone-mediated signaling pathways all *p* > 1.10^−20^, Additional file 12: Fig. S8).

### Transcriptomics of the *L. biglobosa *infection of *B. napus* cotyledons

**Fig. 5.**
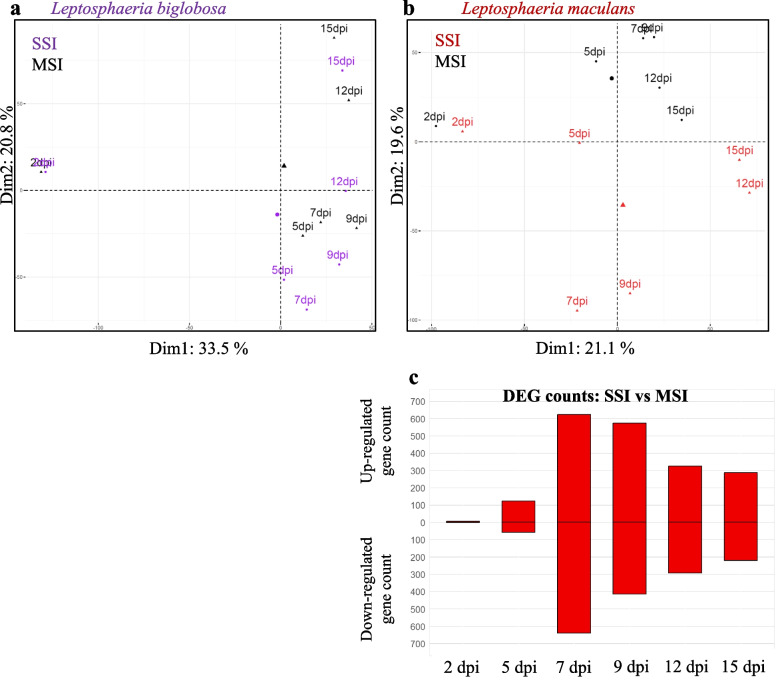
Analysis of *Leptosphaeria maculans* or/and *Leptosphaeria biglobosa* gene expression following inoculation of *Brassica napus*. RNA samples of *Leptosphaeria biglobosa* “brassicae” (Lbb; **a**) or *Leptosphaeria maculans* “brassicae” (Lmb; **b**) were collected at 2, 4, 7, 9, 11, and 15 days post-inoculation (dpi) following inoculation of *Brassica napus* cv. Darmor-*bzh* cotyledons. Single-species inoculations (SSI) were done by inoculating pycnidiospore suspensions of Lmb or Lbb at 10^7^ pycnidiospores.mL^−1^ and mixed species inoculations (MSI) were done by mixing an equal volume of Lmb and Lbb spore suspensions at 10^7^ pycnidiospores.mL^−1^. Principal component analyses (PCA) were done on normalized fungal gene expression (centered Log_2_(FPKM + 1)) of Lbb (**a**) and Lmb (**b**) either in SSI or in MSI. In **a,** samples from Lbb SSI are in purple and samples from MSI are in black. In **b,** samples from Lmb SSI are in red and samples from MSI are in black. Statistical comparisons were done between SSI and MSI samples at each timepoint and for each fungal species. **c** Histogram of the number of differentially expressed genes (DEGs) detected in Lmb following the comparison between SSI and MSI samples at each sampled date (*p* < 0.05, LogFC >|2|). According to the statistical tests, no DEGs were found in Lbb when comparing gene expression following SSI and MSI

According to PCA, Lbb SSI samples were grouped into three clusters (2 dpi; 5 to 9 dpi; 12 to 15 dpi) (Fig. 5a). By contrast, the Lmb samples were scattered and only the 12 and 15 dpi samples were grouped, illustrating the complex waves of expression of this fungus (Fig. 5b). By comparing gene expression between the three Lbb clusters, a total of 1232 DEGs were identified during the kinetics (Additional file 15: Fig. S10). The highest number of DEGs was observed between 2 dpi and all other time points, with 238 genes down-expressed and 578 genes over-expressed, thus highlighting a major transcriptomic reprogramming between 2 and 5 dpi, which is concomitant with the development of Lbb symptoms (Additional file 15: Fig. S10). GO analyses indicated that, at 2 dpi, glycosyl hydrolases (*p* = 2.10^−4^), transport (*p* = 1.10^−2^), and catalytic activities (*p* = 5.10^−4^) were enriched along with four different peptidase activities (metallopeptidase activity: *p* = 3.10^−3^; metallocarboxypeptidase activity: *p* = 5.10^−3^; serine-type (endo)-peptidase activity: *p* = 8.10^−3^; carboxypeptidase activity: *p* = 2.10^−2^) (Additional files 16 and 17: Figs. S11 and S12). Then, two sets of genes were over-expressed departing from 5 dpi. The first one included genes that remained over-expressed all along the cotyledon colonization. This cluster was enriched in heme binding (*p* = 1.10^−2^; Additional file 15: Fig. S10), including six Cytochrome P450 mono-oxygenases, two peroxidases, and one catalase, suggesting an intense detoxification activity. The second one included genes over-expressed 5–9 dpi only. This cluster was enriched in pectate lyase (*p* = 5.10^−4^) and polysaccharide metabolism activities (*p* = 1.10^−6^), suggesting the activation of cell-wall degrading enzymes involved in the necrotrophic behavior of Lbb. Finally, at 12–15 dpi, genes involved in oxido-reduction activities (*p* = 1.10^−5^) were overrepresented including two catalases, five lytic polysaccharide mono-oxygenase (LPMO), and three other mono-oxygenases, one PKS, and one NRPS. Also, processes involving three nitrate reductases and seven alcohol dehydrogenases suggested the activation of a nitrate uptake process that was part of the oxidoreductase activity enrichment at 12–15 dpi. Enrichment analyses of the gene set encoding small secreted proteins (SSP) revealed that they were enriched (*p* = 1.10^−14^) in the DEGs (12%) compared to the set of SSP annotated in the genome (6.8%). By comparing the composition of DEGs in each cluster, a specific enrichment in genes encoding SSPs was detected in the genes upregulated at 5–9 dpi (41% of the cluster; *p* < 2.10^−2^) (Additional file 18: Table S4).

### Transcriptomics of the co-infection on cotyledons

Similarly to what was observed on the plant side, PCA on Lbb gene expression indicated that SSI and MSI samples were closely related at each time point. No DEGs could be detected in Lbb when comparing SSI and MSI (data not shown).

**Fig. 6.**
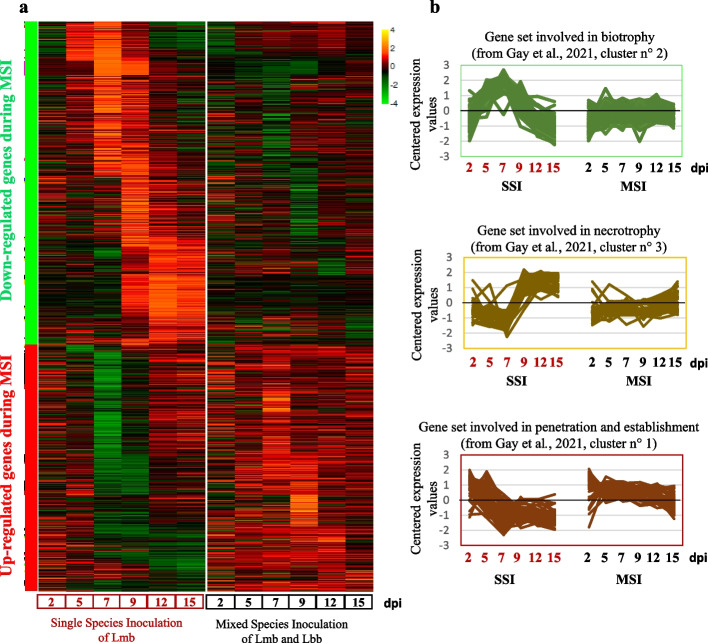
Modification of *Leptosphaeria maculans* “brassicae” gene expression in planta following co-inoculation with *Leptosphaeria biglobosa* “brassicae.” RNA samples were collected at 2, 4, 7, 9, 11, and 15 days post-inoculation (dpi) following inoculation of *Brassica napus* cv. Darmor-*bzh* cotyledons by *Leptosphaeria maculans* “brassicae” (Lmb) or/and *Leptosphaeria biglobosa* “brassicae” (Lbb). Single-species inoculations (SSI) were done by inoculating pycnidiospore suspensions of Lmb or Lbb at 10^7^ pycnidiospores.mL^−1^ and mixed species inoculations (MSI) were done by mixing an equal volume of Lmb and Lbb spore suspensions at 10^7^ pycnidiospores.mL^−1^. Differentially expressed genes (DEGs) were obtained by comparing the Lmb gene expression between SSI and MSI at each date (2, 5, 7, 9, 12, and 15 days post-inoculation; dpi). **a** The heatmap represents the normalized expression (centered Log_2_(FPKM + 1)) of the 2310 DEG set. Genes were grouped into two main categories: the down-expressed gene set in MSI compared to SSI (green bar on the left of the heatmap) or the up-expressed genes in MSI compared to SSI (red bar on the left of the heatmap). **b** Genes belonging to the expression waves highlighted in [28] were extracted from the 2310 DEGs detected in this study. The first three successive expression waves (Cluster 1, 2, and 3, [28]) were found significantly impacted in MSI condition in this study (Additional file 22: Table S5). Changes in expression profiles under MSI condition of genes present in the Cluster1 (“Penetration and establishment”: 2–5 dpi; Cluster2 (“Biotrophy”: 5–9 dpi)) and Cluster3 (“Necrotrophy on cotyledons”: 12–15 dpi)) are shown

Contrasting with what was observed for Lbb, the PCA showed a rapid divergence between Lmb SSI and MSI samples departing from 5 dpi and peaking at 7 dpi (Fig. 5b). No DEGs between SSI and MSI were detected at 2 dpi while a high number of DEGs were detected at 7 dpi and 9 dpi with 1263 and 988 DEGs, respectively (Fig. 5c). In total, 999 Lmb genes were found to be over-expressed and 1311 to be down-expressed following MSI compared to SSI (Fig. 6).

Lmb genes over-expressed in MSI compared to SSI were strongly enriched in catalytic activities at 7, 9, 12, and 15 dpi (Additional file 19: Fig. S13) involving functions such as cutinase activities (*p* = 3.10^−2^; Additional file 19: Fig. S13) or detoxification processes comprising peroxidase (*p* = 2.10^−2^; Additional file 19: Fig. S13) and catalase (*p* = 7.10^−3^; Additional file 19: Fig. S13) genes. ATPase active transmembrane transport was also enriched (*p* = 2.10^−2^) in the genes over-expressed at 7 and 9 dpi and were related to drug efflux (with four ABC multidrug transporter) or to inorganic molecule transport of sodium, potassium, and calcium (Additional file 19: Fig. S13).

The most significantly downregulated Lmb genes in MSI compared to SSI 7 dpi were in GO “Structural constituent of the ribosome” in the “Molecular Function” category and “ribosome biogenesis” in the “Biological Process” category, suggesting an impoverishment in ribosome activity (*p* = 2.10^−43^, Additional files 19, 20 and 21: Figs. S13, S14, and S15) and inhibition of the transcriptional burst usually occurring during the biotrophic stage between 7 and 9 dpi. In addition, genes involved in signal peptide processing (*p* = 6.10^−4^, Additional file 19: Fig. S13) and protein secretion (*p* = 2.10^−4^, Additional file 20: Fig. S14) were enriched among downregulated genes at 7 dpi, suggesting an inhibition of the secretion process, including that of effector proteins. Catalytic activities involved in the necrotrophic stage of the disease on leaves at 12 dpi and 15 dpi were strongly repressed in the presence of Lbb (*p* = 2.10^−3^, Additional file 19: Fig. S13).

### Modification of *L. maculans *in planta expression waves in the presence of *L. biglobosa*

Previous transcriptomic study based on Lmb infection kinetic in SSI (which included cotyledon infection samples used in this study) allowed us to describe 1207 genes involved in the Lmb life cycle distributed in eight expression waves [28]. Comparing this gene set with the DEGs in MSI conditions, we detected that Lmb DEGs in MSI were overrepresented within the three successive waves of gene expression specifically expressed during cotyledon infection in SSI (highlighted in [28]; cluster 1: penetration and establishment, cluster 2: biotrophy, cluster 3: cotyledon necrotrophy, Fig. 6b; Additional file 22: Table S5).

Downregulated genes in MSI were significantly enriched in clusters 2 and 3, while cluster 1 was enriched in genes upregulated in MSI conditions (Fig. 6; Additional file 22: Table S5). The biotrophy cluster (cluster 2) had a higher ratio of DEGs, with 80% of the genes of the cluster under-expressed in Lmb in the presence of Lbb. Its expression profile in MSI no longer showed the expected peak expression at the 5–9 dpi representative of the effector gene expression (Fig. 6b, Additional file 22: Table S5). Similarly, in cluster 3, the absence of the peak of expression occurring from 9 dpi onward was observed for 41% of the genes of this cluster following MSI (Fig. 6b, Additional file 22: Table S5), suggesting that Lmb could not initiate its necrotrophic stage.

Cluster 1, which corresponds to the set of genes expressed early and for a short time, and involved in the penetration and establishment of Lmb [28], was enriched in genes upregulated in MSI conditions (Fig. 6b, Additional file 22: Table S5). In the presence of Lbb, the expression of 50% of genes of this cluster was maintained for the whole of cotyledon colonization (Fig. 6b).

These three consecutive waves of gene expression in SSI were no longer seen following MSI. In contrast, a stagnation of transcriptomic activity was detected between 5 and 15 dpi (Fig. 6).

## Discussion

Using microbiology and transcriptomic approaches, our objective was to dissect the *L. maculans-L. biglobosa-B. napus* interaction to have a better insight into the pathogenic cycle of *L. biglobosa*, and to refine the nature of the tripartite interaction between the plant and the two fungal species.

### *L. biglobosa* pathogenicity and plant tissue colonization

*L. biglobosa* has long been considered a “weakly virulent” pathotype of *L. maculans* before being formally discriminated as a novel species and renamed [31]. Being considered a non-destructive pathogen of rapeseed, and with several “subspecies” that may have different pathogenic abilities [32], studies in the epidemiology, biology, and pathogenic cycle of *L. biglobosa* have been sparse, and sometimes contradictory. In this respect, the LeptoLife project, which generated hundreds of biological samples, including samples from naturally infected Brassica plants ([28], this study), was expected to provide us with extensive knowledge of the Lbb life cycle, as it was the case for Lmb [28]. Unfortunately, and while Lbb was present in all or most samples, the field samples (stems and residues) and inoculated samples (petioles and stems) contained insufficient amounts of Lbb reads to undertake a complete transcriptomic description of the Lbb life cycle. We thus focused on detailed cotyledon infection kinetics to decipher and compare the behavior of the three protagonists during control and mixed inoculations. First, we confirmed that the symptoms developed by the two species differed visually (Fig. 1a) and showed a low Lbb biomass development, measured by qPCR, consistent with studies performed nearly 30 years ago using ELISA quantification of fungal proteins in cotyledons [33]. Despite this limited colonization of tissues, Lbb symptoms appeared much faster than those due to Lmb (5dpi vs. 9dpi) (as already shown for other *L. biglobosa* subspecies such as Lbc [29]). The stagnation of Lbb biomass and the lack of evolution of induced symptoms correlate with stagnation of transcriptomic activity from 5 dpi onward. In contrast, *L. maculans* biomass steadily increases all along the kinetics, with a high biomass increase before the first symptoms appeared, and the setup of a series of successive transcriptomic programs [28]. These observations are consistent with the commonly described necrotrophic and hemibiotrophic lifestyles for Lbb and Lmb, respectively. However, it has to be remembered that *L. biglobosa* also can develop symptomless colonization of tissues of stem and roots (e.g., [34]). The discrepancy between this strictly necrotrophic behavior on leaves and the systemic and symptomless colonization of plant tissues still has to be elucidated.

### Competition (?) between *L. biglobosa *and *L. maculans*

Pathogen-pathogen interactions within the plant may be direct, host mediated, or a combination of the two [2]. The adverse effect of Lbb on Lmb was evident following visual monitoring or biomass measurements of both species, showing that the development of Lmb stopped in the presence of Lbb before primary hyphal development, which led to the complete suppression of Lmb infection. Also, Lbb completely modified the transcriptomic activity of Lmb *in planta*, which became unable to initiate its dynamic gene regulation from 5 to 15 dpi. A series of non-exclusive hypotheses can be proposed to explain these data: (i) direct toxicity of Lbb towards Lmb and/or inhibition of germination of conidia of Lmb in the presence of Lbb, (ii) higher speed of growth of Lbb compared to Lmb, favoring colonization of tissues by the former including a local and quantitative competition for trophic resources, and (iii) adverse effect towards Lmb of the plant defense responses mobilized in response to Lbb infection.

- (i) Direct toxicity of Lbb towards Lmb

The inhibition of Lmb development by Lbb, either in vitro or in planta could be the result of a direct molecular interaction via the specific production by Lbb of molecules with general toxicity or directly targeting Lmb. Metabolite production is widely studied in co-infection assays on distant species. In saprophytic fungi, accumulation of reactive oxygen species, production of toxic metabolites [35], or degrading enzymes could target other competitive species [36]. In fungal phytopathogens, one transcriptomic study suggested that specific effectors were involved in a fungal-fungal interaction during the tripartite infection of barley by *B. graminis* (obligate biotroph) and its antagonist *P. fucculosa* [27]. In our study, we report that the Lbb transcriptomic dynamic was similar in cotyledons whenever Lmb was present or not. This result suggests that no gene sets (including genes involved in metabolites biosynthesis) are specifically activated in Lbb to inhibit Lmb development. Lbb produces secondary metabolites (reviewed by [37]), some of which could be produced constitutively. However, to date, only their phytotoxicity has been evaluated and it is unknown whether some of them could have an antagonist effect against Lmb [37]. In addition, this toxicity would have a dose effect since co-inoculation of Lmb with a lower amount of *L. biglobosa* did not result in growth inhibition of Lmb either in vitro or in planta. We also found no evidence of the production of secondary metabolites among Lbb genes which are over-expressed at 2 or 5dpi. Genes encoding putative proteins involved in the production of secondary metabolites were mostly expressed at the end of the kinetics (12dpi and 15dpi), 10 days after the inhibition of the Lmb development occurred. However, candidate effector genes of unknown function are produced by Lbb at 2 and 5 dpi, which may be involved in competition with other microbes [37]. Another argument against direct antibiosis lies in the lack of detectable effect of inhibition of conidia germination by one species on the other.

- (ii) Higher speed of growth of Lbb compared to Lmb, and competition for trophic resources

Competition between species can be linked to competition for nutrient resources. Nutrient uptake plays a key role in competitive interactions and can be classified as resource-mediated competition for nutrients, which could engender a complete exclusion of one of the competitors. This is described in other antagonistic interactions between fungi or bacteria, in which the competition is mediated, for instance, by the iron chelation by siderophores [38] or through competitions for nutrient resources (amino acids, organic acids, and carbohydrates) [39, 40]. In the literature, *L. biglobosa* is usually described as growing faster than *L. maculans* in axenic conditions as again demonstrated recently on isolates from natural populations [41]. Here, we could not evidence significant differences in growth rate between Lmb and Lbb on V8 agar medium, but a faster growth on MMII minimum medium, suggesting a better ability to use sparse nutrient resources, that may reflect the trophic status of cotyledons. Consistent with this hypothesis, a recent publication suggested Lbb can utilize more carbon sources than Lmb, though it is less efficient than Lmb to use them for its growth [42]. In addition, the Lbb catabolic activity peaks 4 days before that of Lmb [42]. The competition between both species could thus be related to nutrient and resource impoverishment, as suggested by the activation of nutrient uptake in Lbb between 2 and 5 dpi in MSI and SSI. However, if this kind of competition for resources occurs within the Lmb/Lbb complex, this seems to be a very local feature since the interaction between Lbb and Lmb was deleterious for Lmb only during equi-concentrated and concomitant co-inoculation. A second hypothesis involving nutrient availability is that the early induction of the necrotrophic behavior of Lbb on plants could deploy a hostile environment for Lmb via substrate transformation. In SSI, Lmb encompasses a biotrophic stage on living plant tissues during which the nutrient uptake is mediated by the metabolism of the living plant host, i.e., the fungus uses metabolites issued from the plant’s primary metabolism without degrading the plant tissues. The competition between Lmb and Lbb could then be related to the inability of Lmb to feed on necrotic tissues during its biotrophic stage. Yet, Lmb can develop on necrotic tissues, as shown during its two necrotrophic stages on leaves (9–15 dpi) and in the stems [28]. Thus, Lmb possesses genes dedicated to feeding on dead tissues, as also evidenced during its long life as a saprobe on stem residues [28]. The sequential expression waves observed in SSI might not be interchangeable or modulable to adapt its trophic type depending on the substrate changes on leaves induced by Lbb.

- (iii) Adverse effect towards Lmb of the plant defense responses mobilized in response to Lbb infection

In several examples, co-infection of pathogens can lead to positive or negative indirect interaction through the modulation of the plant responses [2, 43]. For instance, the compatible interaction between *Blumeria graminis* and several hosts or the compatible infection of *Albugo candida* on *Brassica juncea* suppresses plant defenses which promote, in both cases, the subsequent infections by avirulent strains or other distant pathogens [44, 45]. During the co-infection in planta or the co-culture in vitro of Lmb and Lbb*,* we observed a drastic impact of Lbb on Lmb, but not the reverse. This result is surprising since, during a compatible interaction, Lmb activates the transcription of dozens of effectors dedicated to suppressing plant defenses during the biotrophic stage [28, 46, 47]. One could have imagined that suppression of plant defense responses by Lmb, mainly when inoculated 2 days before Lbb, would have favored necrotic colonization of the tissue by Lbb, as previously observed by Chen and Fernando [19] for Lbc. This was not the case, with a similar stagnation of Lbb biomass following SSI or when plants were inoculated by both species, whatever the protocol.

Indirect interactions through the induction of local resistance or systemic acquired resistance (SAR) by *L. biglobosa* have been hypothesized to be involved in the competition within the Lmb/Lbb complex. Here we show that the genes encoding plant defense pathways were activated from 5 dpi in response to Lbb or MSI, whereas those genes were activated later in response to Lmb inoculation, i.e., at 9 dpi, during the necrotrophic stage. The MSI with Lbb blocks Lmb at its first pathogenicity step (penetration and establishment in tissues) and prevents it to initiate subsequent pathogenicity programs. Noticeably, Lmb is not killed and can be re-isolated from symptoms following MSI (see also [21]), and it still is transcriptionally active. However, it cannot enter the biotrophy wave, which is characterized by the upregulation of numerous genes encoding effectors postulated to suppress the plant defenses. The involvement of this potential indirect interaction on Lmb during MSI is still unclear, and we still have to disentangle whether, in planta, Lmb is blocked because the plant sets up its responses early in the presence of Lbb, or whether the presence of Lbb acts to suppress Lmb growth (similarly to what is observed in vitro).

### Can *L. biglobosa* be used as a biocontrol agent?

A series of studies proposed that *L. biglobosa*, considered a minor pathogen (or a weakly virulent species), could be used as a biocontrol agent against the damaging *L. maculans* [19–21]. These studies did not investigate interactions between the two species but brought a series of information on the limitation of disease symptoms and plant colonization via the direct response of the plant or systemic acquired resistance following response to *L. biglobosa*. In these studies, the pre-treatments with an analog of Salicylic Acid or with Lbb or Lbc spore suspension both caused a decrease of the Lmb symptom size when inoculated at distant places on the same leaf, on distant leaves or in the field before inoculation with Lmb. Our data and field observations in Europe question this possible use. The Lmb attenuated symptoms [21, 22] or complete exclusion (eMSI, this study) observed in controlled conditions thus contrasts with the Lmb-Lbb cohabitation in the field where leaves are submitted to a constant ascospore shower of both species (along with other fungal pathogens) resulting in numerous entry points for the two species on a single leaf. Co-infection at the same place and time is likely to be very rare under field infections, compared to separated or delayed infection spots, due to leaf surface size and to the length of the period during which primary (ascospores) or secondary (pycnidiospores) inocula are released (from autumn to spring). The lack of or limited impact of Lbb on Lmb when infections are slightly delayed or done with unequal spore concentrations, as observed in our study, probably explains the coexistence of both species in plants all along the life cycle. These results also indicate that protection by *L. biglobosa* from the attacks by *L. maculans *via SAR [20, 22] will probably not be effective in field conditions, at least in Western Europe. Chen and Fernando pointed to the low level of virulence of Lbc in Canada, and its epidemiology (i.e., production of ascospores much later in the season than those of Lmb) to propose pre-treatment by Lbc in the fields before the massive release of Lmb ascospores [19]. The epidemiology of *L. biglobosa* may considerably differ between continents, between countries for the same continent, and between growing seasons for the same country. For example, the timing varied from year to year as a function of climatic conditions in the UK [18]. In many European countries, such as Poland [48] or France [12], ascospores of both species are produced at the same time. In addition, leaf symptoms due to *L. maculans* or *L. biglobosa* are observed at the same time on the same leaves, suggesting a concomitant infection by ascospores of the two species in France [12]. The two species were also found together at the later colonization stage within stem tissues or stem residues, showing that the competition, even if occurring at the leaf level, does not exclude *L. maculans* in the presence of *L. biglobosa* at the stem base [12, 49].

## Conclusions

The tripartite interaction between *L. maculans*,* L. biglobosa*, and *B. napus* is extremely complex and is even complexified *in natura* by the existence of different subspecies of *L. biglobosa* with possibly different pathogenic abilities and the occurrence of numerous other leaf fungal diseases that may interfere with the trio. In addition, our study only considered the fungal interaction involving two isolates which are the sequenced reference of Lbb (G12-14) and Lmb (JN2/JN3) [50], and does not conclude on whether the interactions could have a different outcome should other isolates be evaluated. While JN2 has been recently demonstrated as being representative of the current French population [51], we do not have such information for G12-14. We performed confrontation experiments between JN2 and additional Lbb isolates with similar results, but studies involving interactions between additional couples of isolates would be a valuable addition to the present study.

Our study provides new elements on the biology of *L. biglobosa in planta*. The strong plant response early in the course of infection, the lack of fungal DNA increase with time, and the limited pathogenicity programs that Lbb sets up during infection may question whether Lbb is a necrotrophic pathogen or if it is ill-adapted to *B. napus* and could be more efficient on other Brassicaceae.

The actual contribution of direct competition vs. indirect competition between both species remains elusive at the end of this study, but it indicates that in actual field conditions, the coexistence between both species does not prevent any of the species from reaching the stem and complete its life cycle. As it stands, and given the increasing number of single fungal species being split into closely related species having the same host range (as it was the case for *L. maculans* before 2001 [31]), this study provides a template for similar approaches on multi-pathogen plant interactions.

## Methods

### Plant and fungal materials

The *L. maculans* “brassicae” (Lmb) isolate JN2 (v23.1.2) and the *L. biglobosa* “brassicae” (Lbb) isolate G12-14 were used for inoculations dedicated to RNA extraction; the same isolate constitutively expressing GFP (Lmb) [52] or RFP (Lbb) (this study) were used for all other experiments.

The RFP-Lbb isolate was obtained as follows (modified from [53]): isolate G12-14 was grown on V8-juice agar plates under conditions promoting sporulation [54]. After 14 days of culture, conidia were harvested by flooding the plates with sterile distilled water, followed by filtration. Conidial concentration was adjusted to 10^8^ conidia.mL^−1^ then frozen at − 20°C. *Agrobacterium tumefaciens* strain C58C1 containing pYSKH2::RFP vector was grown in Luria–Bertani (LB) medium containing kanamycin and rifampicin at 50µg.mL^−1^ for 24 h at 28°C. Two mL of the fresh culture were transferred to 18 mL of liquid induction medium (MES 7.8mg.mL^−1^, glucose 10mM, 0.5% glycerol, MM Salt 1X, pH 5.3) containing acetosyringone at 39 µg.mL^−1^. The *A. tumefaciens* cells were then grown at 28°C until the optical density at 600 nm reached 0.6. Equal volumes of the *A. tumefaciens* culture and *L. biglobosa* spore suspension were mixed and 200 µl of the mixture was plated on cellophane sheets placed onto agar transformation medium (MES 7.8 mg.mL^−1^, glucose 5 mM, 0.5% glycerol, MM Salt 1X, acetosyringone 39 µg.mL^−1^, 1.5% agar, pH 5.3) and incubated at 25°C in darkness for 60 h. The cellophane sheets were then transferred onto agar transformation medium plates supplemented with 250 µg.mL^−1^ cefotaxime and 50 µg.mL^−1^ geneticin. Plates were sealed and incubated at 25°C in darkness until fungal colonies were visible. Growing colonies were transferred twice to a minimal medium containing cefotaxime and geneticin and further purified by isolating conidia oozing from a single pycnidium.

The fungi were grown on V8-agar medium and conidia suspensions were produced as described earlier [54] “Darmor-*bzh*,” the reference sequenced cultivar [55] was used in all in planta experiments.

### In vitro competition assays

Ten microliters of pycnidiospore suspensions of Lmb-GFP and/or Lbb-RFP were deposited at the center of 9-cm Petri dishes containing V8-agar or MMII medium. Pycnidiospore suspensions were concentrated at 10^7^ pycnidiospores.mL^−1^ or 10^5^ pycnidiospores.mL^−1^ either in separate spore suspensions for mono-culture of Lmb and Lbb (single-species inoculation; SSI) or mixed in one spore suspension for co-culture (Mixed Species Inoculation; MSI). In the second case, three different procedures were investigated: equi-concentration of 10^7^ pycnidiospores.mL^−1^ (eMSI), a mix of 10^5^ and 10^7^ pycnidiospores.mL^−1^ of Lbb and Lmb, respectively (uMSI), delayed co-inoculations of Lmb and Lbb (10^7^ pycnidiospores.mL^−1^) with Lbb being deposited on the medium 2 days after Lmb (dMSI). The mycelium growth was measured every 2 days from 4 to 14 days of growth. Each plate was observed with a stereomicroscope at 7 days and 14 days, as described below.

The possible inhibition of germination of conidia by one species was investigated by mixing 10^7^ pycnidiospores.mL^−1^ suspension in a liquid medium and examining germination under a microscope after 48 h.

Confrontation experiments between Lmb and Lbb were done by placing the 10^7^ pycnidiospores.mL^−1^ drops 2 cm apart on agar medium. Mycelia were allowed to grow and the confrontation front was observed visually and under the binocular at 9, 11, and 14 dpi. All experiments were repeated at least twice with five Petri dishes per condition.

### Production of biological material

#### In vitro* culture and sampling for RNA extraction*

Lmb or Lbb were grown in vitro on two different media for RNA-sequencing; V8-agar medium or liquid Fries medium. After 7 days of culture, the mycelium was harvested as described previously [28], immediately frozen in liquid nitrogen, and stored at − 80 °C.

#### Cotyledon inoculations and sampling

Cotyledons of 10-day-old plants of cv Darmor-*bzh* were inoculated with deionized water (control), or pycnidiospore suspensions as described previously [56] (SSI). For concomitant co-inoculations (eMSI and uMSI), a mixed suspension of pycnidiospores was inoculated. In addition, delayed co-inoculations of Lmb and Lbb (dMSI) were done.

For RNA-Seq studies, inoculated cotyledon samples were recovered at days 0, 2, 5, 7, 9, 12, and 15 after inoculation. At each time point, eight cotyledons from eight different plants were randomly selected. The plant tissues around the two inoculation sites per cotyledons were cut with a 10-mm disposable punch and the 16 corresponding samples were pooled together in a sterile Falcon tube, immediately frozen in liquid nitrogen, and stored at − 80 °C until extraction. At each time point, two replicates were recovered and the whole experiment was repeated twice.

For biomass quantification, the tissue around the inoculation points (10 mm in diameter) was collected at 2, 4, 7, 9, and 11 dpi, on six randomly selected plants (with four inoculation points per plant). The 24 samples were immediately pooled, frozen in liquid nitrogen, and stored at − 80 °C. At the same time points, two plants were randomly collected for microscopic observations.

### Microscopic observation of infected cotyledons

Each half cotyledon of the two selected plants collected per condition was placed on the lid of a Petri dish and the eight inoculation points were observed with a stereomicroscope (Leica MZ16F, magnification × 60) under brightfield or fluorescent lamp with GFP filter. At each time point, two to four inoculation points were also observed with an optical microscope (Leica DM 5500 B) under the same conditions of light or fluorescence (magnification × 100 or × 400).

### Biomass quantification

Samples were freeze-dried and ground using a Retsch MM300 mixer mill in Eppendorf tubes, with one tungsten carbide bead per tube, for 45 s with 30 oscillations per second. Total DNA was extracted as described by [12]. DNA concentration was measured with a NanoDrop ND-1000 UV–Vis spectrophotometer (NanoDrop, Wilmington, DE, USA).

Fungal DNA was quantified by qPCR as described by [12], using the partial sequence of the *EF1-α* gene for Lmb and of the actin gene for Lbb. For calibration curves, serial dilutions of reference DNA, ranging from 5 × 10^−3^ ng to 5 ng were used in triplicates. In each experiment, each sample was analyzed in three technical replicates.

DNA quantities of Lmb or Lbb between all dates and between all experiments were compared with a Wilcoxon test on R (version 3.4.4) on the average DNA quantity among the three independent biological replicates.

### RNA extraction and sequencing

Leaf discs, cotyledons discs, and freeze-dried cultures of fungal spores or mycelia were ground using a Retsch MM300 mixer mill in Eppendorf tubes, with one tungsten carbide bead per tube, for 45 s with 30 oscillations per second. RNA extractions were performed using the Trizol reagent (Invitrogen, Cergy Pontoise, France) as previously described [57]. RNA-Seq libraries were prepared as reported earlier [50]. Each library was sequenced using 101-bp paired-end reads chemistry on a HiSeq2000 Illumina sequencer.

### Analyses of transcriptomic data

#### Mapping RNA-Seq reads on fungal genomes

Raw RNA-Seq reads were mapped with the STAR software version 020201 [58] on the reference genomes of Lmb (JN3) and Lbb (G12-14) [50]. As Lbb and Lmb isolates are present in the same samples when they are co-inoculated, the mapping parameters have been optimized as follows [28]: we chose a maximum number of mismatches of two and a mapping on a concatenated genome of both species and we allowed an intron size of 10,000 bp. Other parameters have been used as follows: outFilterMultimapNmax: 100; SeedSearchStartLmax: 12; alignSJoverhangMin: 15; alignIntronMin: 10. Then, we selected the properly paired reads in BAM files with Samtools v1.6 [59]. Finally, FeaturesCounts version v1.5.1 [60] was used to quantify the gene expression of uniquely mapped and paired reads.

#### Mapping RNA-Seq reads on the *Brassica napus* genome

RNA-Seq reads were mapped on the *B. napus* V5 genome [55] with the following parameters: outFilterMultimapNmax 6; outFilterMismatchNmax 2; alignIntronMin 10; alignIntronMax 50000; alignMatesGapMax 50000.

#### Sample correlation analysis

The three PCA, made either on Lmb, Lbb, or *B. napus* gene expression data, were done with the FactoRmineR R package [61]. To remove non-expressed genes from the analyses, gene expression had to be greater than two counts in at least one condition. PCA was performed on the scaled Log2(RPKM + 1) expression.

#### Detection of differentially expressed genes

Differential expression analyses were made with the EdgeR package v3.20.9 [62]. Genes with a number of reads > 30 in at least one condition were kept for statistical analysis. Samples were normalized by the TMM method. The data were fitted to a negative binomial generalized linear model by the glmFIt function. Then, the glmLRT function was used to compare the gene expression using appropriate coefficients and contrasts. Fungal gene expression (Lmb or Lbb) was compared between SSI and MSI for each of the six time points of the kinetics. In *B. napus*, treatments (co-inoculation or mono-inoculation) were compared to the control cotyledons “inoculated” with water. As no *B. napus* genes were detected differentially expressed between Lbb SSI and MSI, the contrasts were adapted to compare the Lbb SSI and MSI together against the mock-inoculated water control samples. In each comparison, genes were selected as differentially expressed with a LogFC > 2 and a *p* < 0.05.

#### Gene ontology enrichment analysis

The plugin Bingo of the Cytoscape software v3.5.1 [63] was used to conduct the Gene Ontology (GO) enrichment analysis. For each gene, the GO identifier was generated by the BLAST2GO software. Significant enrichments in “Molecular Function,” “Biological Process,” and “Cellular Component” were detected with a hypergeometrical test by comparing the proportion of a given GO function in the gene subset of interest to its proportion in the whole protein set of Lmb, Lbb, or *B. napus*. The *p*-values were corrected for multiple testing using the FDR method.

### Supplementary Information

**Additional file 1:** **Fig. S1.** Growth profiles of *Leptosphaeria maculans* ‘brassicae’ (Lmb) isolate JN2-GFP, *Leptosphaeria biglobosa* ‘brassicae’ (Lbb) isolate G12-14-RFP, and mixes of the two species together on agar medium. The isolates were inoculated as 10^7^ spores.mL^−1^ pycnidiospore suspension (i, Lmb; ii, Lbb; iii, Lmb + Lbb), as 10^5^ spores.mL^−1^ pycnidiospore suspension for Lbb only, or as a mix of 10^7^ spores.mL^−1^ Lmb pycnidiospore suspension + 10^5^ spores.mL^−1^ Lbb pycnidiospore suspension (iv), and grown in the dark on V-8 agar medium (a, b) or MMII agar medium (c, d). (a, c) Diameters were measured for 14 days of growth, (b, d) morphology of the colonies at 11 dpi.

**Additional file 2:** **Fig. S2.** Visualisation of *Leptosphaeria maculans *‘brassicae’ (Lmb)-GFP and *Leptosphaeria biglobosa* ‘brassicae’ (Lbb)-RFP mycelia during growth of mixed species on agar medium. The inoculum mix consisted of a mix of 10^7^ spores mL^−1^ Lmb pycnidiospore suspension + 10^5^ spores mL^−1^ Lbb pycnidiospore suspension. Pictures were taken after four (a, b) and seven (c) days of growth on MMII agar medium (a, c) or V-8 agar medium (b). For each panel : left picture, bright field; center picture : GFP-expressing Lmb; right picture, RFP- expressing Lbb.

**Additional file 3:** **Fig. S3.** Confrontation experiments between *Leptosphaeria maculans* ‘brassicae’ (Lmb) and *Leptosphaeria biglobosa* ‘brassicae’ (Lbb). Each species was deposited as a droplet containing 10^7^ spores.mL^−1^ ca. two cm apart on V-8 agar medium and allowed to grow for 14 days. Pictures of the Petri dishes were taken at 4, 7, 9, 11, 14 days post-inoculation.

**Additional file 4:** **Table S1.** Results of read mapping from RNA-Seq of 52 samples corresponding to infection of cotyledons of *Brassica napus* by *Leptosphaeria maculans* ‘brassicae’, *Leptosphaeria biglobosa* ‘brassicae’ and both species together.

**Additional file 5:** **Fig. S4.** Monitoring of *Leptosphaeria maculans* ‘brassicae’ (Lmb) and *Leptosphaeria biglobosa* ‘brassicae’ (Lbb) *Brassica napus *tissue colonization following various regimes of cotyledon infections. In the case of Single Species Inoculation (SSI), the cotyledons of *B. napus *were inoculated with (a) a pycnidiospore suspension of Lmb (transgenic isolate JN2 expressing GFP) at 10^7^ pycnidiospores.mL^−1^ or (b) with a pycnidiospore suspension of Lbb (isolate G12-14) at 10^7^ pycnidiospores.mL^−1^. In the cases of Mixed Species Inoculation (MSI), co-inoculations were made by inoculating a mixed suspension of pycnidiospores of both Lmb and Lbb species in equal quantity (10^7^ pycnidiospores.mL^−1^ each) (eMSI (c)) or Lmb at 10^7^ pycnidiospores.mL^−1^ and Lbb 100-times less concentrated at 10^5^ pycnidiospores.mL^−1^ (uMSI (d)). Lastly, a delayed MSI (dMSI) was done with an addition of Lbb inoculum two days after the inoculation of Lmb (e), both inocula being at 10^7^ pycnidiospores.mL^−1^. The cotyledons were collected at six different time points (4,7,9,11,14 days post inoculation; dpi). Observations were made using a stereomicroscope under brightfield (upper panels) or under a GFP filter (magnification: X60). A photonic microscope was used to make observations with higher magnification factor under brightfield and GFP filter (X100 and X400). The pictures are representative observations made on eight infection points with three biological replicates.

**Additional file 6:** **Table S2.** Biomass quantification of *Leptosphaeria maculans* ‘brassicae’ (Lmb) and *Leptosphaeria biglobosa* ‘brassicae’ (Lbb) following various regimes of cotyledon infections.a DNA (in ng) of Lmb and/or Lbb were quantified by qPCR during six regimes of cotyledon infections: SSI 7: Single Species Inoculation at 10^7^ pycnidiospores.mL^−1^ (control conditions); SSI 5: Single Species Inoculation at 10^5^ pycnidiospores.mL^−1^ ; dMSI : Delayed Mixed Species Inoculation (Lbb 10^7^ pycnidiospores.mL^−1^ was inoculated 2 days after Lmb 10^7^ pycnidiospores.mL^−1^); uMSI : unequal Mixed Species Inoculation in which both Lbb and Lmb were inoculated simultaneously but Lbb was inoculated at lower concentration (10^5^ pycnidiospores.mL^−1^) ; eMSI : equal Mixed Species Inoculation. Both Lbb and Lmb were inoculated simultaneously and at the same concentration (10^7^ pycnidiospores.mL^−1^). *At each time point, DNA quantities of Lmb and Lbb were compared to the control condition of SSI at 10^7^ pycnidiospores.mL^−1^ for Lmb and SSI at 10^7^ or 10^5^ pycnidiospores.mL^−1^ for Lbb. The asterisks (*) represent a significant difference with *p* < 0.05 (see legend of Fig. 2)..

**Additional file 7:** **Fig. S5.** Proportion of RNA-Seq reads assigned to* Leptosphaeria maculans* ‘brassicae’ (Lmb) and *Leptosphaeria biglobosa* ‘brassicae’ (Lbb) during Single Species Inoculation (SSI) or Mixed Species Inoculation (MSI) of *Brassica napus* cotyledons. (a) and (b), percentages of reads assigned to Lmb (a) or Lbb (b) among the total number of reads at the six sampling timepoints (2,5,7,9,12,15 days post-inoculation, dpi). (c) Cumulated percentage of reads of the two fungal species following MSI. (d) Relative proportion of fungal reads assigned to Lmb or Lbb following MSI. For each time point, data from two biological replicates are shown as dark and pale color (red and pink bars for Lmb, blue and pale blue bars for Lbb, black and grey for MSI).

**Additional file 8:** **Table S3.** Correlation of gene expression between biological replicates following Single Species Inoculation of cotyledons of *Brassica napus *with *Leptosphaeria maculans* ‘brassicae’ (Lmb), *Leptosphaeria biglobosa *(Lbb) or following Mixed Species Inoculation of Lmb and Lbb.

**Additional file 9:** **Text S1.** Variability in RNA-Seq replicates.

**Additional file 10:** **Fig. S6. **Top twenty enrichments of the Gene Ontology category “Biological Process” detected in the gene sets of *Brassica napus* upregulated during Lmb SSI and/or Lbb SSI. ‘Lmb SSI’, top twenty enrichments found in genes upregulated compared to the mock inoculated plant following Single Species Inoculation (SSI) with Lmb. ‘Lbb SSI + MSI’, top twenty enrichments found following Lbb SSI and Mixed Species Inoculation (Lmb + Lbb). ‘Lmb SSI and ‘Lbb SSI + MSI’, top twenty enrichments found as a common response to all inoculation procedures. For the tree gene sets, enrichments analyses were done using a hypergeometrical test with the Cytoscape tool Bingo. The y axis indicates the overrepresented Biological Process terms. The x axis represents the resulting -Log_10_(FDR) of the enrichment test. The numbers in the boxes indicate the number of genes assigned to the corresponding Biological Process in the cluster (left) and the total number of genes associated to this Biological Process term in the whole gene set (right).

**Additional file 11:** **Fig. S7.** Top twenty enrichments of the Gene Ontology category “Molecular Function” detected in the gene sets of *Brassica napus *up-regulated during Lmb SSI and/or Lbb SSI. ‘Lmb SSI’, top twenty enrichments found in genes up-regulated compared to the mock inoculated plant following Single Species Inoculation (SSI) with Lmb. ‘Lbb SSI + MSI’, top twenty enrichments found following Lbb SSI and Mixed Species Inoculation (Lmb + Lbb). ‘Lmb SSI and ‘Lbb SSI + MSI’, top twenty enrichments found as a common response to all inoculation procedures. For the tree gene sets, enrichments analyses were done using an hypergeometrical test with the Cytoscape tool Bingo. The y axis indicates the overrepresented Molecular Function terms. The x axis represents the resulting -Log_10_(FDR) of the enrichment test. The numbers in the boxes indicate the number of genes assigned to the corresponding Molecular Function in the cluster (left) and the total number of genes associated to this Molecular Function term in the whole gene set (right).

**Additional file 12:** **Fig. S8.** Top twenty enrichments of the Gene Ontology category “Biological Process” detected in the gene sets of *Brassica napus* down-regulated during Lmb SSI and/or Lbb SSI. ‘Lmb SSI’, top twenty enrichments found in genes down-regulated compared to the mock inoculated plant following Single Species Inoculation (SSI) with Lmb. ‘Lbb SSI + MSI’, top twenty enrichments found following Lbb SSI and Mixed Species Inoculation (Lmb + Lbb). ‘Lmb SSI and ‘Lbb SSI + MSI’, top twenty enrichments found as a common response to all inoculation procedures. For the tree gene sets, enrichments analyses were done using an hypergeometrical test with the Cytoscape tool Bingo. The y axis indicates the overrepresented Biological Process terms. The x axis represents the resulting -Log_10_(FDR) of the enrichment test. The numbers in the boxes indicate the number of genes assigned to the corresponding Biological Process in the cluster (left) and the total number of genes associated to this Biological Process term in the whole gene set (right).

**Additional file 13:** **Fig. S9.** Top twenty enrichments of the Gene Ontology category “Molecular Function” detected in the gene sets of *Brassica napus *down-regulated during Lmb SSI and/or Lbb SSI.‘Lmb SSI’, top twenty enrichments found in genes down-regulated compared to the mock inoculated plant following Single Species Inoculation (SSI) with Lmb. ‘Lbb SSI + MSI’, top twenty enrichments found following Lbb SSI and Mixed Species Inoculation (Lmb + Lbb). ‘Lmb SSI and ‘Lbb SSI + MSI’, top twenty enrichments found as a common response to all inoculation procedures. For the tree gene sets, enrichments analyses were done using an hypergeometrical test with the Cytoscape tool Bingo. The y axis indicates the overrepresented Molecular Function terms. The x axis represents the resulting -Log_10_(FDR) of the enrichment test. The numbers in the boxes indicate the number of genes assigned to the corresponding Molecular Function in the cluster (left) and the total number of genes associated to this Molecular Function term in the whole gene set (right).

**Additional file 14:** **Text S2.** Specific response to Lmb or Lbb in *Brassica napus *gene expression.

**Additional file 15:** **Fig. S10.** Expression profiles of the 1,233 genes of *Leptosphaeria biglobosa* ‘brassicae’ (Lbb) detected as differentially expressed during the 15-days infection of *Brassica napus* cotyledons either following single species inoculation (SSI) or when inoculated as a mix (MSI) with *Leptosphaeria maculans* ‘brassicae’. Differentially expressed genes (DEGs) were detected using three different comparisons on samples obtained from the SSI inoculation of Lbb : 2 dpi vs 5-7-9-12-15 dpi (816 genes, black bar on the left of the heatmap), 5-7-9 dpi vs. 2-12-15 dpi (130 genes, orange bar on the left of the heatmap) and 12-15 dpi vs 2-5-7-9 dpi (286 genes, blue bar on the left of the heatmap). Gene raw counts of Lbb in SSI and MSI conditions were Log_2_(FPKM +1) transformed and then centered. The heatmap represents the normalized expression of DEGs detected on SSI and their expression are also shown during MSI. Genes annotated in the Small Secreted Protein repertoire are shown on the immediate left of the heatmap as black bars. Five categories of the “Molecular Function” Gene Ontology have a significant enrichment and are identified by colored bars on the left of the heatmap (Catalytic activities in black; Chitin binding in cyan; Glycosyl hydrolase activity in blue; Pectate lyase activity in red; Transmembrane transport activity in brown).

**Additional file 16:** **Fig. S11.** Detection of Gene Ontology enrichments (“Molecular Function” category) among *Leptosphaeria biglobosa *‘brassicae’ down-regulated gene set (green) or up-regulated gene set (red) during infection of cotyledons of *Brassica napus*. For each of the three stages depicted in Fig. 3 (2 days post-inoculation (dpi), 5-7-9 dpi, and 12-15 dpi), GO enrichment in the set of genes up-regulated (red) or down-regulated (green) was identified using a hypergeometrical test with the Cytoscape tool Bingo. The y axis indicates the terms overrepresented in the Molecular function category. The x axis represents the -Log_10_(FDR) of the enrichment test. The numbers in the boxes indicate the number of genes assigned to the corresponding Molecular Function in the cluster (left) and the total number of genes associated to this Molecular Function term in the whole genome gene set (right).

**Additional file 17:** **Fig. S12.** Detection of Gene Ontology enrichments (“Biological process” category) among *Leptosphaeria biglobosa *‘brassicae’ down-regulated gene set (green) or up-regulated gene set (red) during infection of cotyledons of *Brassica napus*. For each of the three stages depicted in Fig. 3 (2 days post-inoculation (dpi), 5-7-9dpi, and 12-15 dpi), GO enrichment in the set of genes up-regulated (red) or down-regulated (green) was identified using a hypergeometrical test with the Cytoscape tool Bingo. The y axis indicates the terms overrepresented in the Biological Process category. The x axis represents the -Log_10_(FDR) of the enrichment test. The numbers in the boxes indicate the number of genes assigned to the corresponding Biological Process in the cluster (left) and the total number of genes associated to this Biological Process term in the whole genome gene set (right).

**Additional file 18:** **Table S4.** Enrichment analyses of the proportion of genes encoding Small Secreted Proteins (SSP) in the Differentially expressed gene (DEG) sets detected in *Leptosphaeria biglobosa* ‘brassicae’ during cotyledon infections. ^a^ To detect if genes encoding SSPs were over-represented in DEGs, their proportion was compared to those of SSPs in the entire gene set of Lbb by a Chi-Squared test (** : *p*-value < 0.001). ^b^ To detect if a specific enrichment of genes encoding SSPs was found in each group of DEGs (2 dpi; 5-9 dpi; 12-15 dpi), their proportion was compared to the proportion of SSPs in the total DEGs set by a Chi-Squared test (** : *p*-value < 0.001, *: *p*-value < 0.05).

**Additional file 19:** **Fig. S13.** Detection of Gene Ontology enrichments (“Molecular Function” category) among *Leptosphaeria maculans* ‘brassicae’ (Lmb) down-regulated gene set (green) or up-regulated gene set (red) during Mixed Species Inoculation (MSI) compared to Single Species Inoculation (SSI). For each time point of the kinetics of infection (5, 7, 9, 12 or 15 days post-inoculation-dpi), GO enrichment in the set of genes up-regulated (red) or down-regulated (green) in Lmb during MSI compared to SSI was identified using a hypergeometrical test with the Cytoscape tool Bingo. The y axis indicates the terms overrepresented in the Molecular Function category. The x axis represents the -Log_10_(FDR) of the enrichment test. The numbers in the boxes indicate the number of genes assigned to the corresponding Molecular Function in the cluster (left) and the total number of genes associated to this Biological Process term in the whole genome gene set (right).

**Additional file 20:** **Fig. S14.** Detection of Gene Ontology enrichments (“Biological Process” category) among *Leptosphaeria maculans* ‘brassicae’ (Lmb) down-regulated gene set (green) or up-regulated gene set (red) during Mixed Species Inoculation (MSI) compared to Single Species Inoculation (SSI). For each time point of the kinetics of infection (5, 7, 9, 12 or 15 days post-inoculation-dpi), GO enrichment in the set of genes up-regulated (red) or down-regulated (green) in Lmb during MSI compared to SSI was identified using a hypergeometrical test with the Cytoscape tool Bingo. The y axis indicates the terms overrepresented in the Biological Process category. The x axis represents the -Log_10_(FDR) of the enrichment test. The numbers in the boxes indicate the number of genes assigned to the corresponding Biological Process in the cluster (left) and the total number of genes associated to this Biological Process term in the whole genome gene set (right).

**Additional file 21:** **Fig. S15.** Detection of Gene Ontology enrichments (“Cellular Component” category) among *Leptosphaeria maculans* ‘brassicae’ (Lmb) down-regulated gene set (green) or up-regulated gene set (red) during Mixed Species Inoculation (MSI) compared to Single Species Inoculation (SSI). For each time point of the kinetics of infection (5, 7, 9, 12 or 15 days post-inoculation-dpi), GO enrichment in the set of genes up-regulated (red) or down-regulated (green) in Lmb during MSI compared to SSI was identified using a hypergeometrical test with the Cytoscape tool Bingo. The y axis indicates the terms overrepresented in the Cellular Component category. The x axis represents the -Log_10_(FDR) of the enrichment test. The numbers in the boxes indicate the number of genes assigned to the corresponding Cellular Component in the cluster (left) and the total number of genes associated to this Cellular Component term in the whole genome gene set (right).

**Additional file 22:** **Table S5.** Modification of *Leptosphaeria maculans* ‘brassicae’ (Lmb) gene expression during plant infection in the presence of Leptosphaeria biglobosa ‘brassicae’ (Lbb; Mixed Species Inoculation : MSI). ^a^ Total number of genes in the Lmb genome and total number of genes specifically expressed during the infectious cycle [28]. ^b^ The proportion of Differentially expressed genes (DEGs) in MSI (17.7% of the total gene set) was compared to the proportion of DEGs in MSI in the 1,203 gene set (51.8%) by Chi-Squared test (**: *p* < 0.001, *: *p* < 0.05). ^c^ As described by Gay et al. [28], the 1207 genes specifically expressed by Lmb during plant infection are grouped into eight expression waves representative of the fungal lifestyle and/or of specific organs colonized. ^d^ To detect which expression waves was significantly impacted by the MSI conditions, the proportion of DEGs in each wave was compared to the total proportion of DEG in the 1,207 genes involved in the infectious cycle using a Chi-Squared test (**: *p* < 0.001, *: *p* < 0.05). Three waves (1, 2 and 3) were highly enriched in DEGs during MSI condition (in bold). Two clusters (5 and 6 corresponding to stem infection) were underrepresented in the DEGs in MSI conditions. ^e^ To detect if genes in clusters 1, 2 and 3 trended to be up- or downregulated in MSI conditions, a second Chi-Squared test (**: *p* < 0.001, *: *p* < 0.05) was done by comparing the proportion of down- and upregulated genes in each cluster in MSI to the total amount of down- and upregulated genes among the 1,207 genes set. ^f^ Nd : not done, statistical tests were only performed on overrepresented clusters.

## Data Availability

RNA-seq data are available from NCBI under BioProject accessions PRJEB21682 [ERX2086273- ERX2086296] and PRJEB47779 [ERX6466175- ERX6466212]. All data submitted via different bioprojects are available under an umbrella bioproject PRJEB63477 [64].

## Abbreviations

ABC transporter: ATP-binding cassette transporter
ATP: Adenosine triphosphate
DEGs: Differentially expressed genes
dpi: Days post-inoculation
ELISA: Enzyme-Linked ImmunoSorbent Assay
FC: Fold change
FDR: False discovery rate
GFP/RFP: Green/red fluorescent protein
GO: Gene Ontology
Lbb: *Leptosphaeria biglobosa *“brassicae”
Lbc: *Leptosphaeria biglobosa* “canadensis”
Lmb: *Leptosphaeria maculans* “brassicae”
LPMO: Lytic polysaccharide mono-oxygenase
MSI: Mixed Species Inoculations
dMSI: Mixed Species Inoculations with an equal number of spores for each species, but Lbb spores were added two days after those of Lmb
eMSI: Mixed Species Inoculations with an equal number of spores for each species
uMSI: Mixed Species Inoculations with an unequal number of spores for each species (100-x less spores for Lbb compared to Lmb)
PAMP: Pathogen-associated molecular pattern
PCA: Principal component analysis
qPCR: Quantitative polymerase chain reaction
RPKM: Reads per kilobase of transcript per million reads mapped
SAR: Systemic acquired resistance
SSI: Single species inoculation
SSP: Small secreted protein
