# Supplementary material for: Location and timing govern tripartite interactions of fungal phytopathogens and host in the stem canker species complex
Source: BMC Biol. 2023 Nov 7;21:247. doi: 10.1186/s12915-023-01726-8 (PMC10631019; doi:10.1186/s12915-023-01726-8)
Supplement: Supplementary file 1 — Additional file 1: Fig. S1. Growth profiles of Leptosphaeria maculans ‘brassicae’ (Lmb) isolate JN2-GFP, Leptosphaeria biglobosa ‘brassicae’ (Lbb) isolate G12-14-RFP, and mixes of the two species together on agar medium. The isolates were inoculated as 107 spores.mL−1 pycnidiospore suspension (i, Lmb; ii, Lbb; iii, Lmb + Lbb), as 105 spores.mL−1 pycnidiospore suspension for Lbb only, or as a mix of 107 spores.mL−1 Lmb pycnidiospore suspension + 105 spores.mL−1 Lbb pycnidiospore suspension (iv), and grown in the dark on V-8 agar medium (a, b) or MMII agar medium (c, d). (a, c) Diameters were measured for 14 days of growth, (b, d) morphology of the colonies at 11 dpi. [file 12915_2023_1726_MOESM1_ESM.pptx]

## Slide 1
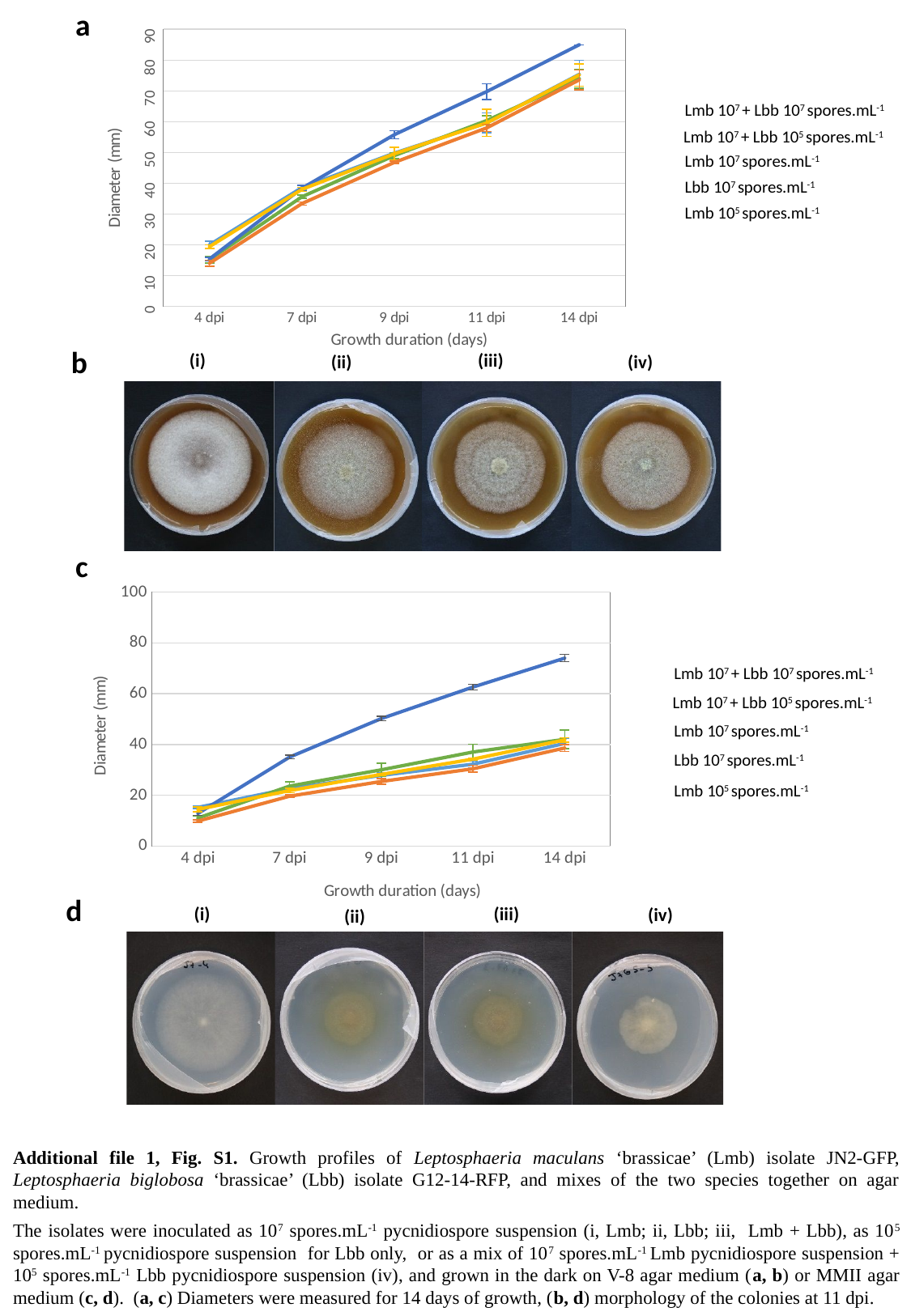

a
### Chart
| Category | Lmb 10^7 + Lbb 10^7 spores/mL | Lmb 10^7 + Lbb 10^5 spores/mL | Lmb 10^7 spores/mL | Lbb 10^7 spores/mL | Lmb 10^5 spores/mL |
|---|---|---|---|---|---|
| 4 dpi | 20.0 | 15.2 | 15.4 | 19.4 | 14.0 |
| 7 dpi | 38.6 | 35.6 | 38.4 | 38.0 | 33.4 |
| 9 dpi | 49.8 | 49.0 | 55.8 | 49.6 | 46.8 |
| 11 dpi | 59.6 | 60.4 | 69.8 | 59.6 | 58.0 |
| 14 dpi | 75.4 | 74.0 | 85.0 | 75.2 | 73.6 |Lmb 107 + Lbb 107 spores.mL-1
Lmb 107 + Lbb 105 spores.mL-1
Lmb 107 spores.mL-1
Lbb 107 spores.mL-1
Lmb 105 spores.mL-1
b
(i)
(iii)
(ii)
(iv)
c
### Chart
| Category | Lmb 10^7 + Lbb 10^7 spores/mL | Lmb 10^7 + Lbb 10^5 spores/mL | Lmb 10^7 spores/mL | Lbb 10^7 spores/mL | Lmb 10^5 spores/mL |
|---|---|---|---|---|---|
| 4 dpi | 15.2 | 11.0 | 12.8 | 14.4 | 9.8 |
| 7 dpi | 22.6 | 23.6 | 35.0 | 21.8 | 19.6 |
| 9 dpi | 27.8 | 30.0 | 50.2 | 28.2 | 25.4 |
| 11 dpi | 32.2 | 37.0 | 62.6 | 34.2 | 30.4 |
| 14 dpi | 40.4 | 42.0 | 74.0 | 41.8 | 38.6 |Lmb 107 + Lbb 107 spores.mL-1
Lmb 107 + Lbb 105 spores.mL-1
Lmb 107 spores.mL-1
Lbb 107 spores.mL-1
Lmb 105 spores.mL-1
d
(i)
(iii)
(iv)
(ii)
Additional file 1, Fig. S1. Growth profiles of Leptosphaeria maculans ‘brassicae’ (Lmb) isolate JN2-GFP, Leptosphaeria biglobosa ‘brassicae’ (Lbb) isolate G12-14-RFP, and mixes of the two species together on agar medium.
The isolates were inoculated as 107 spores.mL-1 pycnidiospore suspension (i, Lmb; ii, Lbb; iii, Lmb + Lbb), as 105 spores.mL-1 pycnidiospore suspension for Lbb only, or as a mix of 107 spores.mL-1 Lmb pycnidiospore suspension + 105 spores.mL-1 Lbb pycnidiospore suspension (iv), and grown in the dark on V-8 agar medium (a, b) or MMII agar medium (c, d). (a, c) Diameters were measured for 14 days of growth, (b, d) morphology of the colonies at 11 dpi.
