# Supplementary material for: Location and timing govern tripartite interactions of fungal phytopathogens and host in the stem canker species complex
Source: BMC Biol. 2023 Nov 7;21:247. doi: 10.1186/s12915-023-01726-8 (PMC10631019; doi:10.1186/s12915-023-01726-8)
Supplement: Supplementary file 2 — Additional file 2: Fig. S2. Visualisation of Leptosphaeria maculans ‘brassicae’ (Lmb)-GFP and Leptosphaeria biglobosa ‘brassicae’ (Lbb)-RFP mycelia during growth of mixed species on agar medium. The inoculum mix consisted of a mix of 107 spores mL−1 Lmb pycnidiospore suspension + 105 spores mL−1 Lbb pycnidiospore suspension. Pictures were taken after four (a, b) and seven (c) days of growth on MMII agar medium (a, c) or V-8 agar medium (b). For each panel : left picture, bright field; center picture : GFP-expressing Lmb; right picture, RFP- expressing Lbb. [file 12915_2023_1726_MOESM2_ESM.pptx]

## Slide 1
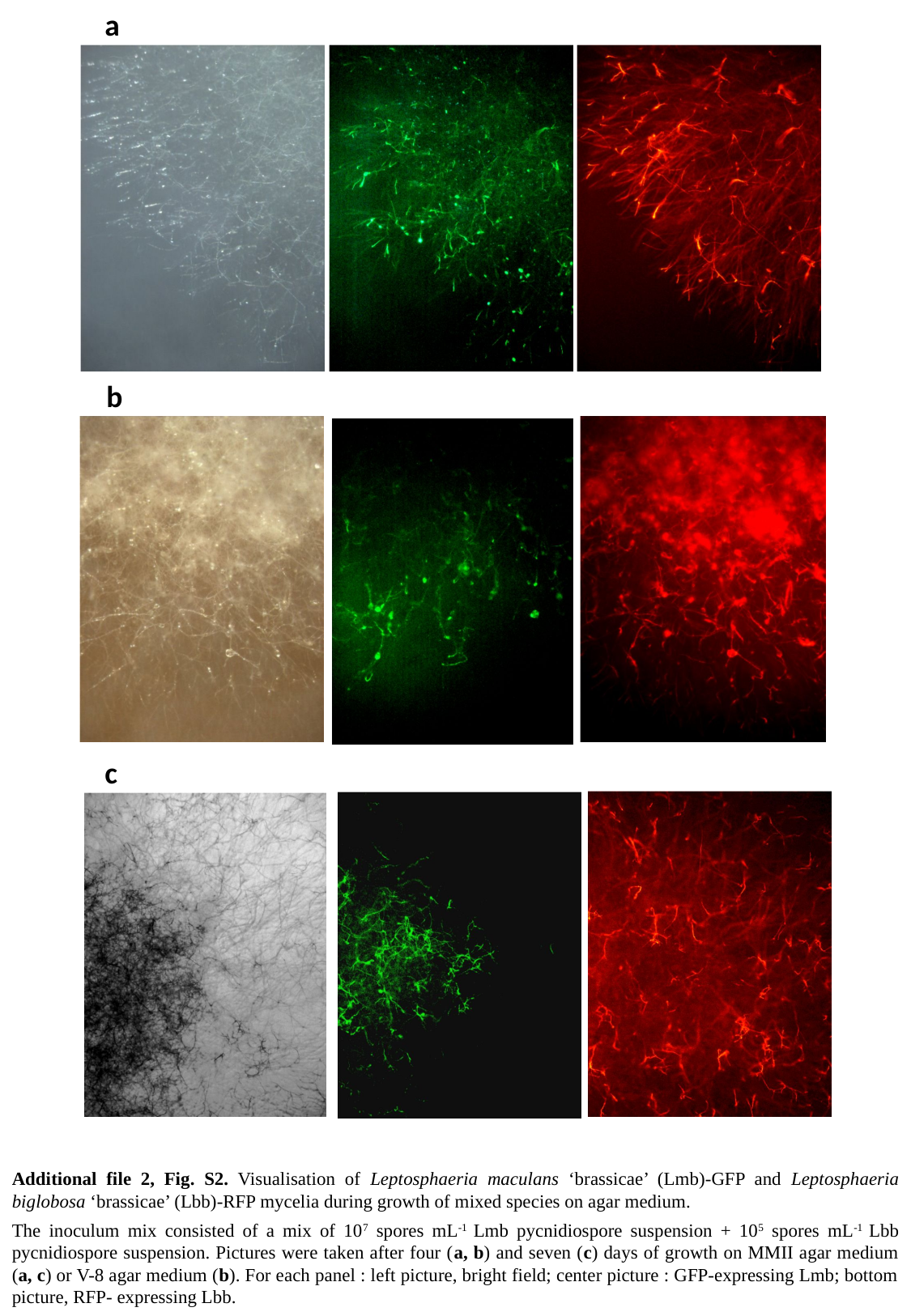

a
b
c
Additional file 2, Fig. S2. Visualisation of Leptosphaeria maculans ‘brassicae’ (Lmb)-GFP and Leptosphaeria biglobosa ‘brassicae’ (Lbb)-RFP mycelia during growth of mixed species on agar medium.
The inoculum mix consisted of a mix of 107 spores mL-1 Lmb pycnidiospore suspension + 105 spores mL-1 Lbb pycnidiospore suspension. Pictures were taken after four (a, b) and seven (c) days of growth on MMII agar medium (a, c) or V-8 agar medium (b). For each panel : left picture, bright field; center picture : GFP-expressing Lmb; bottom picture, RFP- expressing Lbb.
