# Supplementary material for: Location and timing govern tripartite interactions of fungal phytopathogens and host in the stem canker species complex
Source: BMC Biol. 2023 Nov 7;21:247. doi: 10.1186/s12915-023-01726-8 (PMC10631019; doi:10.1186/s12915-023-01726-8)
Supplement: Supplementary file 3 — Additional file 3: Fig. S3. Confrontation experiments between Leptosphaeria maculans ‘brassicae’ (Lmb) and Leptosphaeria biglobosa ‘brassicae’ (Lbb). Each species was deposited as a droplet containing 107 spores.mL−1 ca. two cm apart on V-8 agar medium and allowed to grow for 14 days. Pictures of the Petri dishes were taken at 4, 7, 9, 11, 14 days post-inoculation. [file 12915_2023_1726_MOESM3_ESM.pptx]

## Slide 1
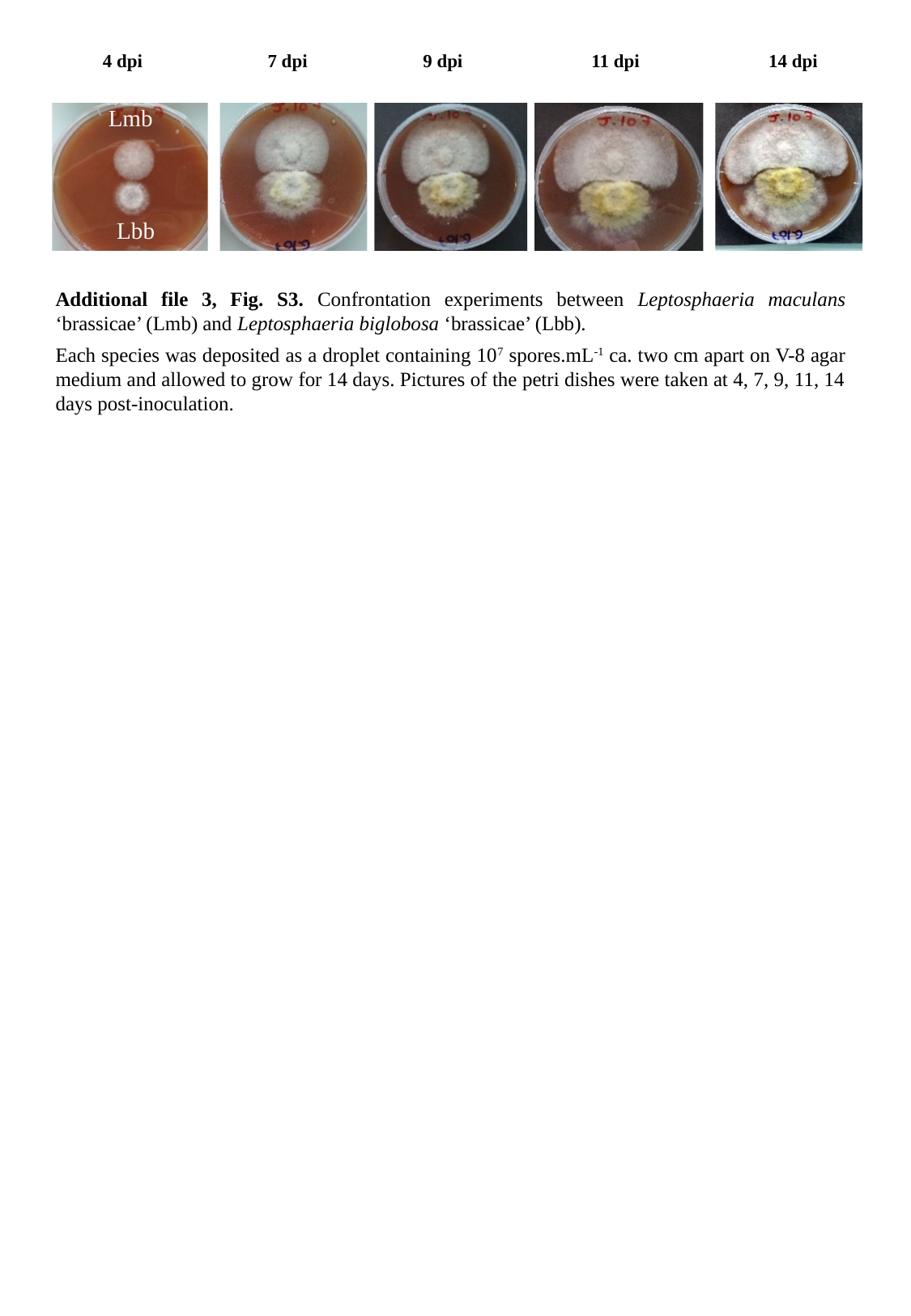

4 dpi
7 dpi
9 dpi
11 dpi
14 dpi
Lmb
Lbb
Additional file 3, Fig. S3. Confrontation experiments between Leptosphaeria maculans ‘brassicae’ (Lmb) and Leptosphaeria biglobosa ‘brassicae’ (Lbb).
Each species was deposited as a droplet containing 107 spores.mL-1 ca. two cm apart on V-8 agar medium and allowed to grow for 14 days. Pictures of the petri dishes were taken at 4, 7, 9, 11, 14 days post-inoculation.
