# Supplementary material for: Location and timing govern tripartite interactions of fungal phytopathogens and host in the stem canker species complex
Source: BMC Biol. 2023 Nov 7;21:247. doi: 10.1186/s12915-023-01726-8 (PMC10631019; doi:10.1186/s12915-023-01726-8)
Supplement: Supplementary file 5 — Additional file 5: Fig. S4. Monitoring of Leptosphaeria maculans ‘brassicae’ (Lmb) and Leptosphaeria biglobosa ‘brassicae’ (Lbb) Brassica napus tissue colonization following various regimes of cotyledon infections. In the case of Single Species Inoculation (SSI), the cotyledons of B. napus were inoculated with (a) a pycnidiospore suspension of Lmb (transgenic isolate JN2 expressing GFP) at 107 pycnidiospores.mL−1 or (b) with a pycnidiospore suspension of Lbb (isolate G12-14) at 107 pycnidiospores.mL−1. In the cases of Mixed Species Inoculation (MSI), co-inoculations were made by inoculating a mixed suspension of pycnidiospores of both Lmb and Lbb species in equal quantity (107 pycnidiospores.mL−1 each) (eMSI (c)) or Lmb at 107 pycnidiospores.mL−1 and Lbb 100-times less concentrated at 105 pycnidiospores.mL−1 (uMSI (d)). Lastly, a delayed MSI (dMSI) was done with an addition of Lbb inoculum two days after the inoculation of Lmb (e), both inocula being at 107 pycnidiospores.mL−1. The cotyledons were collected at six different time points (4,7,9,11,14 days post inoculation; dpi). Observations were made using a stereomicroscope under brightfield (upper panels) or under a GFP filter (magnification: X60). A photonic microscope was used to make observations with higher magnification factor under brightfield and GFP filter (X100 and X400). The pictures are representative observations made on eight infection points with three biological replicates. [file 12915_2023_1726_MOESM5_ESM.pptx]

## Slide 1
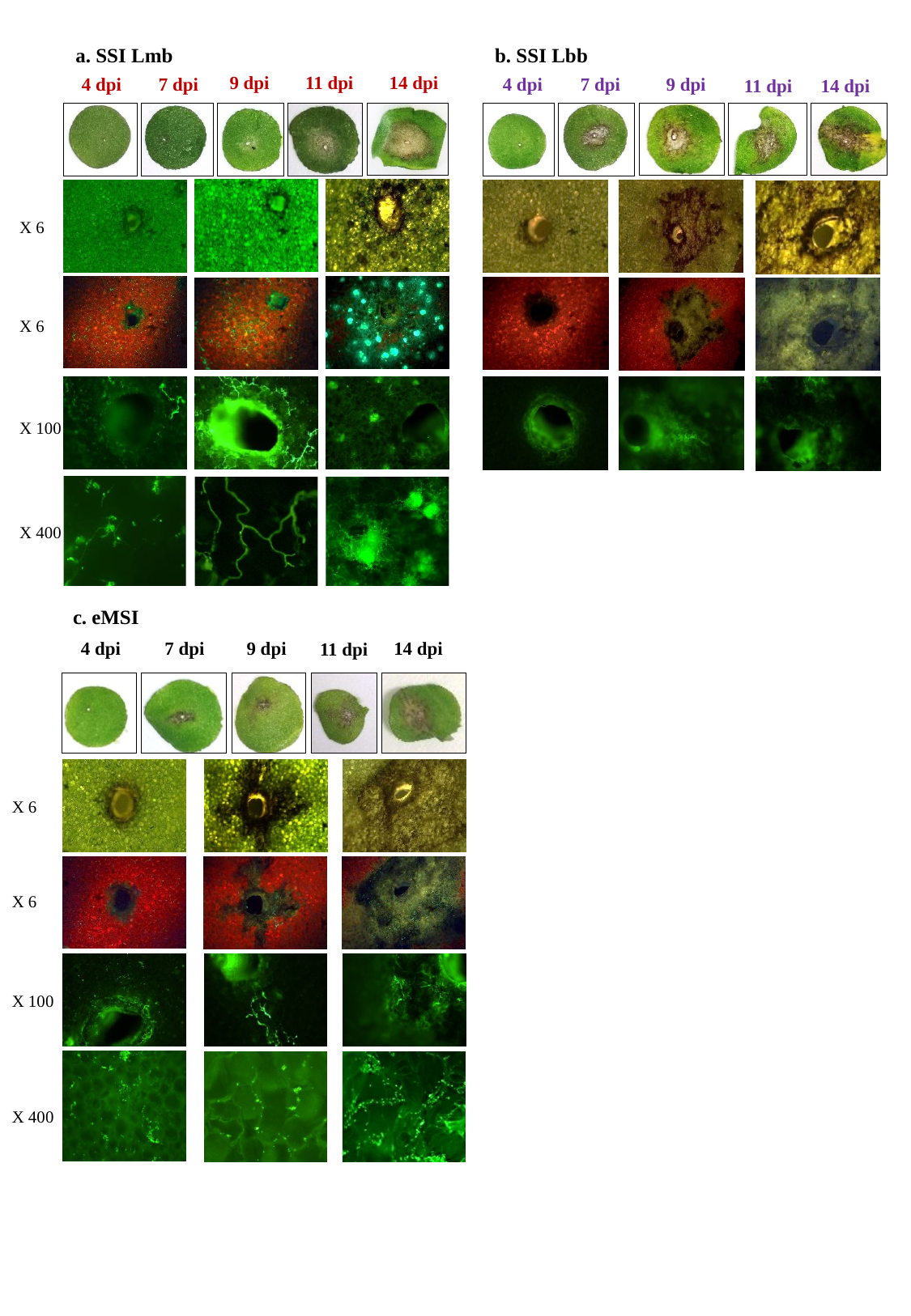

a. SSI Lmb
b. SSI Lbb
14 dpi
4 dpi
7 dpi
9 dpi
11 dpi
4 dpi
7 dpi
9 dpi
14 dpi
11 dpi
X 6
X 6
X 100
X 400
c. eMSI
4 dpi
7 dpi
9 dpi
14 dpi
11 dpi
X 6
X 6
X 100
X 400

## Slide 2
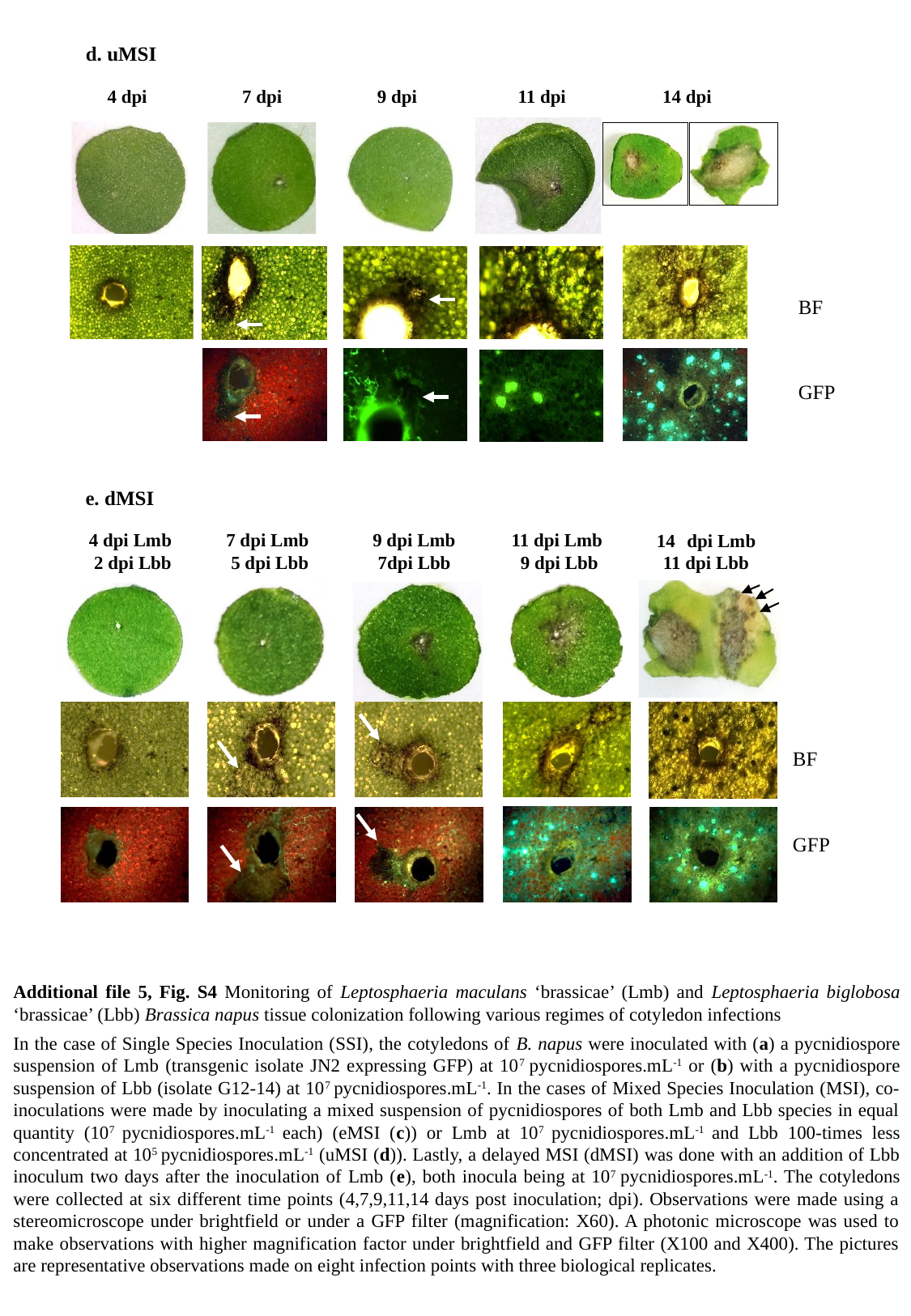

d. uMSI
4 dpi
7 dpi
11 dpi
14 dpi
9 dpi
BF
GFP
e. dMSI
dpi Lmb
11 dpi Lbb
4 dpi Lmb
2 dpi Lbb
7 dpi Lmb
5 dpi Lbb
9 dpi Lmb 7dpi Lbb
11 dpi Lmb
9 dpi Lbb
BF
GFP
Additional file 5, Fig. S4 Monitoring of Leptosphaeria maculans ‘brassicae’ (Lmb) and Leptosphaeria biglobosa ‘brassicae’ (Lbb) Brassica napus tissue colonization following various regimes of cotyledon infections
In the case of Single Species Inoculation (SSI), the cotyledons of B. napus were inoculated with (a) a pycnidiospore suspension of Lmb (transgenic isolate JN2 expressing GFP) at 107 pycnidiospores.mL-1 or (b) with a pycnidiospore suspension of Lbb (isolate G12-14) at 107 pycnidiospores.mL-1. In the cases of Mixed Species Inoculation (MSI), co-inoculations were made by inoculating a mixed suspension of pycnidiospores of both Lmb and Lbb species in equal quantity (107 pycnidiospores.mL-1 each) (eMSI (c)) or Lmb at 107 pycnidiospores.mL-1 and Lbb 100-times less concentrated at 105 pycnidiospores.mL-1 (uMSI (d)). Lastly, a delayed MSI (dMSI) was done with an addition of Lbb inoculum two days after the inoculation of Lmb (e), both inocula being at 107 pycnidiospores.mL-1. The cotyledons were collected at six different time points (4,7,9,11,14 days post inoculation; dpi). Observations were made using a stereomicroscope under brightfield or under a GFP filter (magnification: X60). A photonic microscope was used to make observations with higher magnification factor under brightfield and GFP filter (X100 and X400). The pictures are representative observations made on eight infection points with three biological replicates.
