# Supplementary material for: Location and timing govern tripartite interactions of fungal phytopathogens and host in the stem canker species complex
Source: BMC Biol. 2023 Nov 7;21:247. doi: 10.1186/s12915-023-01726-8 (PMC10631019; doi:10.1186/s12915-023-01726-8)
Supplement: Supplementary file 6 — Additional file 6: Table S2. Biomass quantification of Leptosphaeria maculans ‘brassicae’ (Lmb) and Leptosphaeria biglobosa ‘brassicae’ (Lbb) following various regimes of cotyledon infections.a DNA (in ng) of Lmb and/or Lbb were quantified by qPCR during six regimes of cotyledon infections: SSI 7: Single Species Inoculation at 107 pycnidiospores.mL−1 (control conditions); SSI 5: Single Species Inoculation at 105 pycnidiospores.mL−1 ; dMSI : Delayed Mixed Species Inoculation (Lbb 107 pycnidiospores.mL−1 was inoculated 2 days after Lmb 107 pycnidiospores.mL−1); uMSI : unequal Mixed Species Inoculation in which both Lbb and Lmb were inoculated simultaneously but Lbb was inoculated at lower concentration (105 pycnidiospores.mL−1) ; eMSI : equal Mixed Species Inoculation. Both Lbb and Lmb were inoculated simultaneously and at the same concentration (107 pycnidiospores.mL−1). *At each time point, DNA quantities of Lmb and Lbb were compared to the control condition of SSI at 107 pycnidiospores.mL−1 for Lmb and SSI at 107 or 105 pycnidiospores.mL−1 for Lbb. The asterisks (*) represent a significant difference with p < 0.05 (see legend of Fig. 2).. [file 12915_2023_1726_MOESM6_ESM.pptx]

## Slide 1
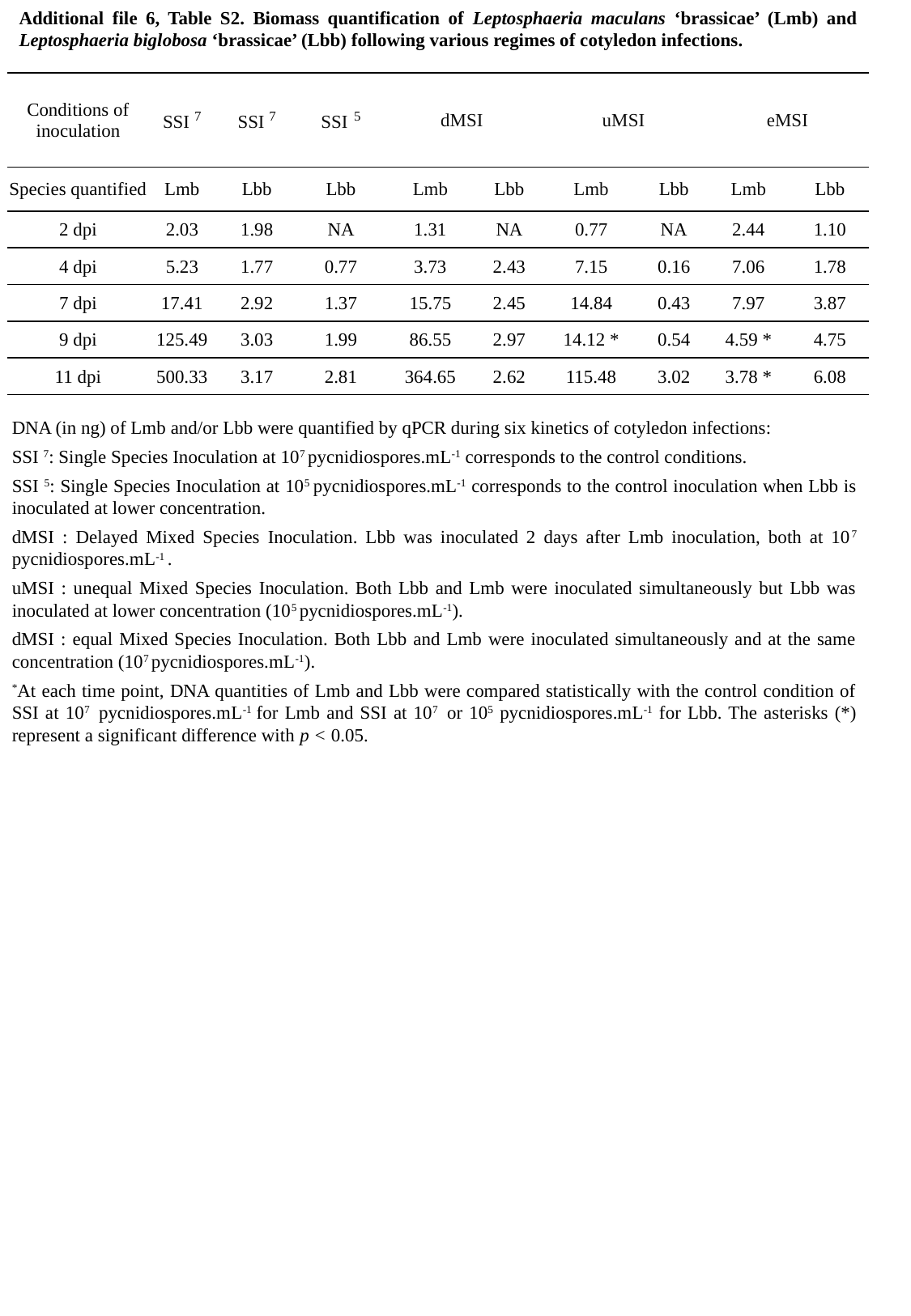

Additional file 6, Table S2. Biomass quantification of Leptosphaeria maculans ‘brassicae’ (Lmb) and Leptosphaeria biglobosa ‘brassicae’ (Lbb) following various regimes of cotyledon infections.
| Conditions of inoculation | SSI 7 | SSI 7 | SSI 5 | dMSI | | uMSI | | eMSI | |
| --- | --- | --- | --- | --- | --- | --- | --- | --- | --- |
| Species quantified | Lmb | Lbb | Lbb | Lmb | Lbb | Lmb | Lbb | Lmb | Lbb |
| 2 dpi | 2.03 | 1.98 | NA | 1.31 | NA | 0.77 | NA | 2.44 | 1.10 |
| 4 dpi | 5.23 | 1.77 | 0.77 | 3.73 | 2.43 | 7.15 | 0.16 | 7.06 | 1.78 |
| 7 dpi | 17.41 | 2.92 | 1.37 | 15.75 | 2.45 | 14.84 | 0.43 | 7.97 | 3.87 |
| 9 dpi | 125.49 | 3.03 | 1.99 | 86.55 | 2.97 | 14.12 \* | 0.54 | 4.59 \* | 4.75 |
| 11 dpi | 500.33 | 3.17 | 2.81 | 364.65 | 2.62 | 115.48 | 3.02 | 3.78 \* | 6.08 |
DNA (in ng) of Lmb and/or Lbb were quantified by qPCR during six kinetics of cotyledon infections:
SSI 7: Single Species Inoculation at 107 pycnidiospores.mL-1 corresponds to the control conditions.
SSI 5: Single Species Inoculation at 105 pycnidiospores.mL-1 corresponds to the control inoculation when Lbb is inoculated at lower concentration.
dMSI : Delayed Mixed Species Inoculation. Lbb was inoculated 2 days after Lmb inoculation, both at 107 pycnidiospores.mL-1 .
uMSI : unequal Mixed Species Inoculation. Both Lbb and Lmb were inoculated simultaneously but Lbb was inoculated at lower concentration (105 pycnidiospores.mL-1).
dMSI : equal Mixed Species Inoculation. Both Lbb and Lmb were inoculated simultaneously and at the same concentration (107 pycnidiospores.mL-1).
*At each time point, DNA quantities of Lmb and Lbb were compared statistically with the control condition of SSI at 107 pycnidiospores.mL-1 for Lmb and SSI at 107 or 105 pycnidiospores.mL-1 for Lbb. The asterisks (*) represent a significant difference with p < 0.05.
