# Supplementary material for: Location and timing govern tripartite interactions of fungal phytopathogens and host in the stem canker species complex
Source: BMC Biol. 2023 Nov 7;21:247. doi: 10.1186/s12915-023-01726-8 (PMC10631019; doi:10.1186/s12915-023-01726-8)
Supplement: Supplementary file 7 — Additional file 7: Fig. S5. Proportion of RNA-Seq reads assigned to Leptosphaeria maculans ‘brassicae’ (Lmb) and Leptosphaeria biglobosa ‘brassicae’ (Lbb) during Single Species Inoculation (SSI) or Mixed Species Inoculation (MSI) of Brassica napus cotyledons. (a) and (b), percentages of reads assigned to Lmb (a) or Lbb (b) among the total number of reads at the six sampling timepoints (2,5,7,9,12,15 days post-inoculation, dpi). (c) Cumulated percentage of reads of the two fungal species following MSI. (d) Relative proportion of fungal reads assigned to Lmb or Lbb following MSI. For each time point, data from two biological replicates are shown as dark and pale color (red and pink bars for Lmb, blue and pale blue bars for Lbb, black and grey for MSI). [file 12915_2023_1726_MOESM7_ESM.pptx]

## Slide 1
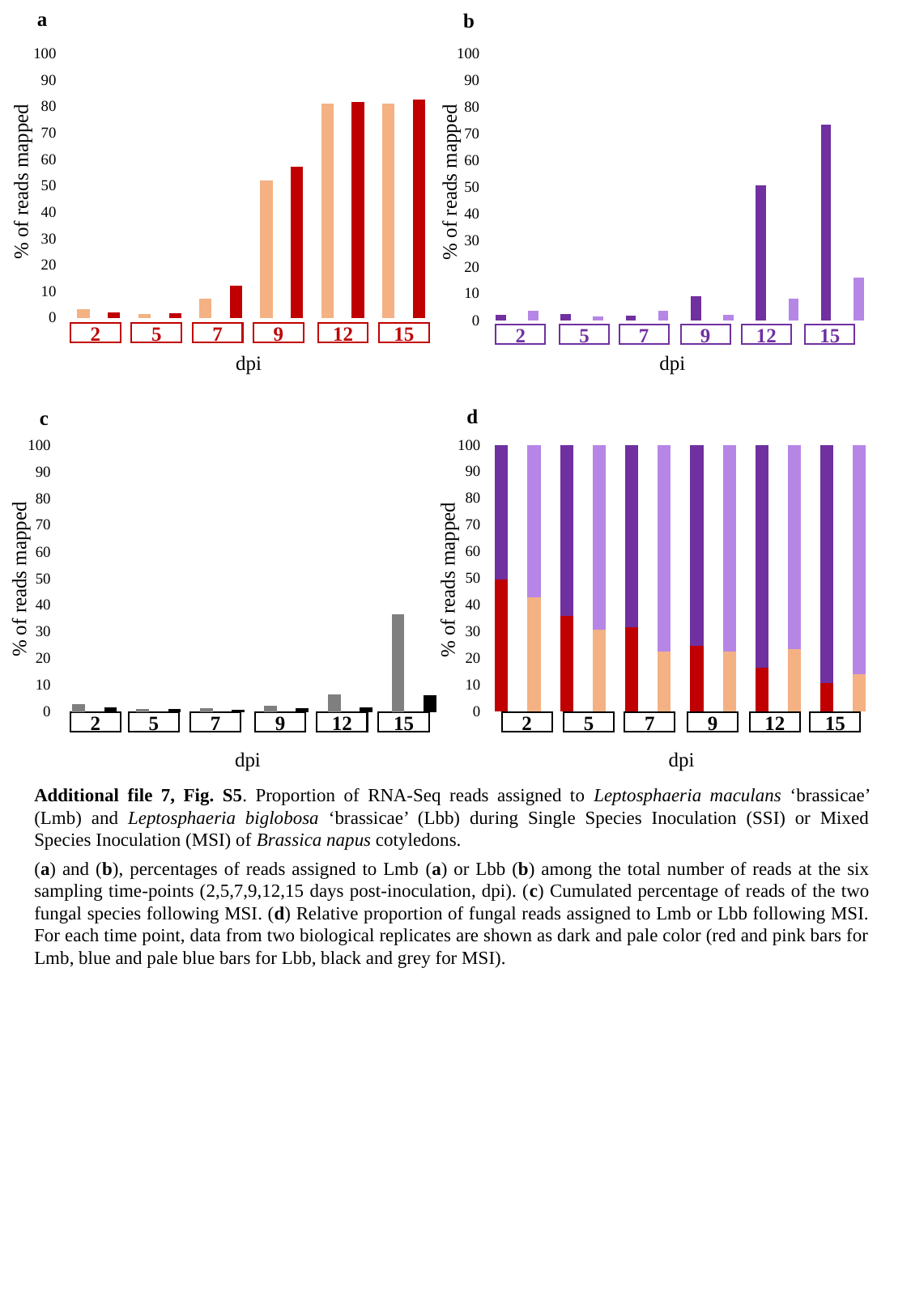

a
b
### Chart
| Category | |
|---|---|
### Chart
| Category | |
|---|---|% of reads mapped
% of reads mapped
2
5
7
9
12
15
2
5
7
9
12
15
dpi
dpi
d
c
### Chart
| Category | | |
|---|---|---|
### Chart
| Category | |
|---|---|% of reads mapped
% of reads mapped
2
5
7
9
12
15
2
5
7
9
12
15
dpi
dpi
Additional file 7, Fig. S5. Proportion of RNA-Seq reads assigned to Leptosphaeria maculans ‘brassicae’ (Lmb) and Leptosphaeria biglobosa ‘brassicae’ (Lbb) during Single Species Inoculation (SSI) or Mixed Species Inoculation (MSI) of Brassica napus cotyledons.
(a) and (b), percentages of reads assigned to Lmb (a) or Lbb (b) among the total number of reads at the six sampling time-points (2,5,7,9,12,15 days post-inoculation, dpi). (c) Cumulated percentage of reads of the two fungal species following MSI. (d) Relative proportion of fungal reads assigned to Lmb or Lbb following MSI. For each time point, data from two biological replicates are shown as dark and pale color (red and pink bars for Lmb, blue and pale blue bars for Lbb, black and grey for MSI).
