# Supplementary material for: Location and timing govern tripartite interactions of fungal phytopathogens and host in the stem canker species complex
Source: BMC Biol. 2023 Nov 7;21:247. doi: 10.1186/s12915-023-01726-8 (PMC10631019; doi:10.1186/s12915-023-01726-8)
Supplement: Supplementary file 8 — Additional file 8: Table S3. Correlation of gene expression between biological replicates following Single Species Inoculation of cotyledons of Brassica napus with Leptosphaeria maculans ‘brassicae’ (Lmb), Leptosphaeria biglobosa (Lbb) or following Mixed Species Inoculation of Lmb and Lbb. [file 12915_2023_1726_MOESM8_ESM.pptx]

## Slide 1
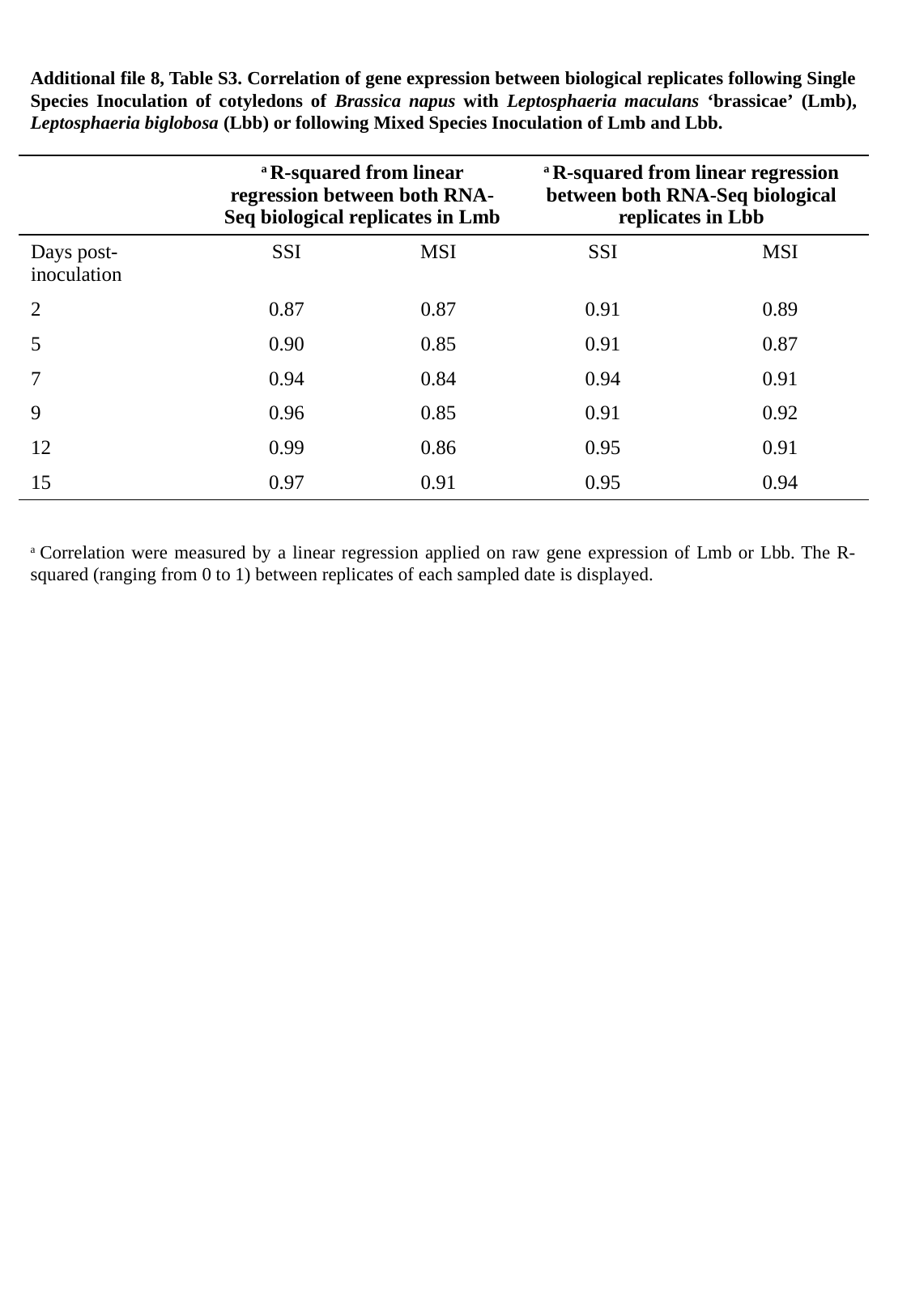

Additional file 8, Table S3. Correlation of gene expression between biological replicates following Single Species Inoculation of cotyledons of Brassica napus with Leptosphaeria maculans ‘brassicae’ (Lmb), Leptosphaeria biglobosa (Lbb) or following Mixed Species Inoculation of Lmb and Lbb.
| | a R-squared from linear regression between both RNA-Seq biological replicates in Lmb | | a R-squared from linear regression between both RNA-Seq biological replicates in Lbb | |
| --- | --- | --- | --- | --- |
| Days post-inoculation | SSI | MSI | SSI | MSI |
| 2 | 0.87 | 0.87 | 0.91 | 0.89 |
| 5 | 0.90 | 0.85 | 0.91 | 0.87 |
| 7 | 0.94 | 0.84 | 0.94 | 0.91 |
| 9 | 0.96 | 0.85 | 0.91 | 0.92 |
| 12 | 0.99 | 0.86 | 0.95 | 0.91 |
| 15 | 0.97 | 0.91 | 0.95 | 0.94 |
a Correlation were measured by a linear regression applied on raw gene expression of Lmb or Lbb. The R-squared (ranging from 0 to 1) between replicates of each sampled date is displayed.
