# Supplementary material for: Location and timing govern tripartite interactions of fungal phytopathogens and host in the stem canker species complex
Source: BMC Biol. 2023 Nov 7;21:247. doi: 10.1186/s12915-023-01726-8 (PMC10631019; doi:10.1186/s12915-023-01726-8)
Supplement: Supplementary file 9 — Additional file 9: Text S1. Variability in RNA-Seq replicates. [file 12915_2023_1726_MOESM9_ESM.docx]

**Additional file 9, S1 Text. Variability in RNA-Seq replicates.**

The proportion of RNA-Seq reads assigned to Lmb or Lbb following SSI on cotyledons (Additional file 7: S5a,b Fig) firstly revealed differences between replicates for Lbb SSI: while the proportion of reads assigned to Lmb was comparable at a given time between replicates, the proportion of reads assigned to Lbb was variable between replicates with differences between samples as high as 15.91% and 73.25% of the reads at 15 dpi (Additional files 4 and 7: S1 Table, S5a,b Fig**)**. Despite this variability, the linear regression of gene expression between replicates showed that the r-squared at each time point were always higher than 0.90 (Additional file 8: S3 Table), which validated the use of comparative statistical tests.
