# Supplementary material for: Location and timing govern tripartite interactions of fungal phytopathogens and host in the stem canker species complex
Source: BMC Biol. 2023 Nov 7;21:247. doi: 10.1186/s12915-023-01726-8 (PMC10631019; doi:10.1186/s12915-023-01726-8)
Supplement: Supplementary file 12 — Additional file 12: Fig. S8. Top twenty enrichments of the Gene Ontology category “Biological Process” detected in the gene sets of Brassica napus down-regulated during Lmb SSI and/or Lbb SSI. ‘Lmb SSI’, top twenty enrichments found in genes down-regulated compared to the mock inoculated plant following Single Species Inoculation (SSI) with Lmb. ‘Lbb SSI + MSI’, top twenty enrichments found following Lbb SSI and Mixed Species Inoculation (Lmb + Lbb). ‘Lmb SSI and ‘Lbb SSI + MSI’, top twenty enrichments found as a common response to all inoculation procedures. For the tree gene sets, enrichments analyses were done using an hypergeometrical test with the Cytoscape tool Bingo. The y axis indicates the overrepresented Biological Process terms. The x axis represents the resulting -Log10(FDR) of the enrichment test. The numbers in the boxes indicate the number of genes assigned to the corresponding Biological Process in the cluster (left) and the total number of genes associated to this Biological Process term in the whole gene set (right). [file 12915_2023_1726_MOESM12_ESM.pptx]

## Slide 1
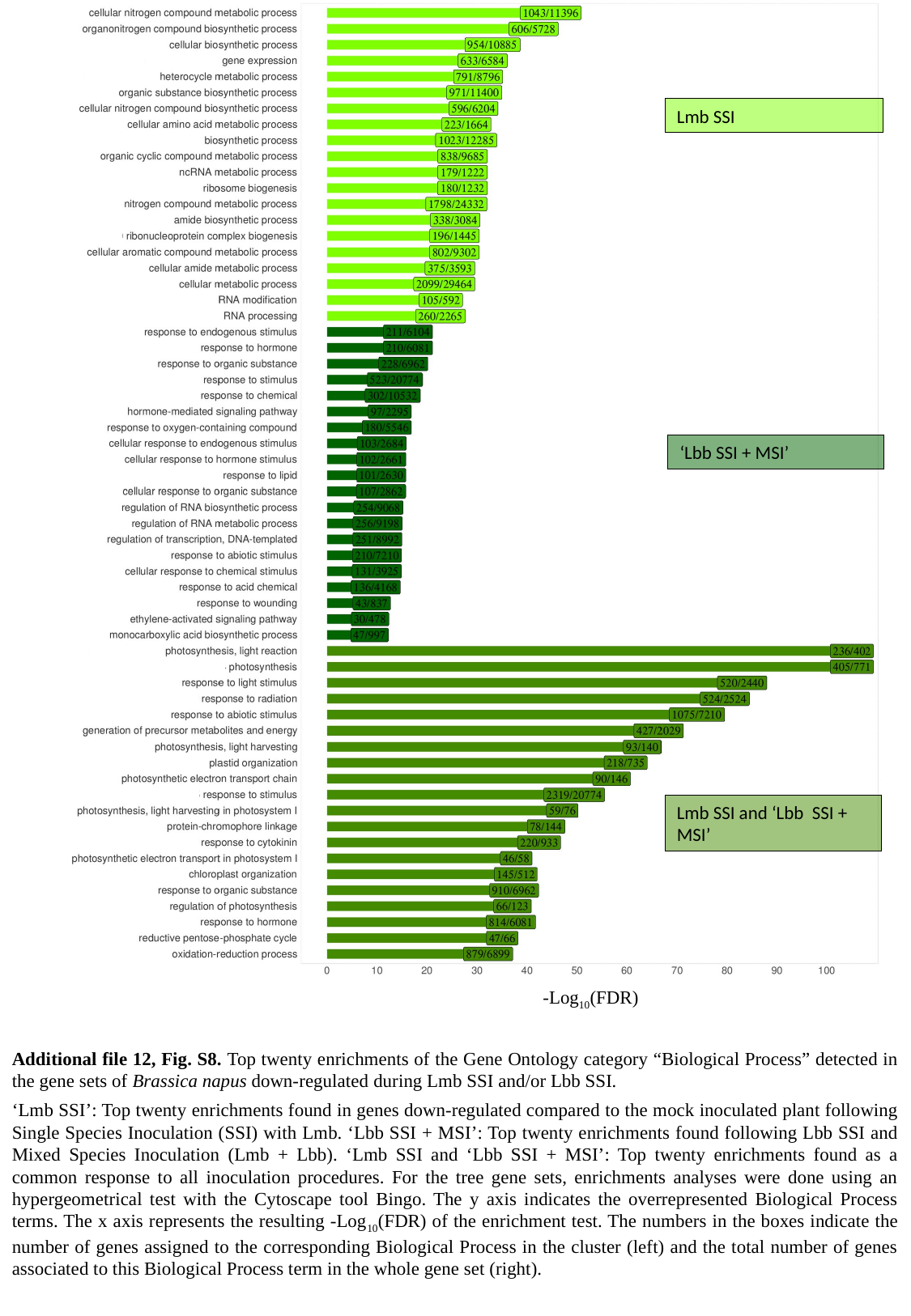

Lmb SSI
‘Lbb SSI + MSI’
Lmb SSI and ‘Lbb SSI + MSI’
-Log10(FDR)
Additional file 12, Fig. S8. Top twenty enrichments of the Gene Ontology category “Biological Process” detected in the gene sets of Brassica napus down-regulated during Lmb SSI and/or Lbb SSI.
‘Lmb SSI’: Top twenty enrichments found in genes down-regulated compared to the mock inoculated plant following Single Species Inoculation (SSI) with Lmb. ‘Lbb SSI + MSI’: Top twenty enrichments found following Lbb SSI and Mixed Species Inoculation (Lmb + Lbb). ‘Lmb SSI and ‘Lbb SSI + MSI’: Top twenty enrichments found as a common response to all inoculation procedures. For the tree gene sets, enrichments analyses were done using an hypergeometrical test with the Cytoscape tool Bingo. The y axis indicates the overrepresented Biological Process terms. The x axis represents the resulting -Log10(FDR) of the enrichment test. The numbers in the boxes indicate the number of genes assigned to the corresponding Biological Process in the cluster (left) and the total number of genes associated to this Biological Process term in the whole gene set (right).
