# Supplementary material for: Location and timing govern tripartite interactions of fungal phytopathogens and host in the stem canker species complex
Source: BMC Biol. 2023 Nov 7;21:247. doi: 10.1186/s12915-023-01726-8 (PMC10631019; doi:10.1186/s12915-023-01726-8)
Supplement: Supplementary file 14 — Additional file 14: Text S2. Specific response to Lmb or Lbb in Brassica napus gene expression. [file 12915_2023_1726_MOESM14_ESM.docx]

**Additional file 14, S2 Text. Specific response to Lmb or Lbb in B.napus gene expression**

A total of 26,852 genes of *B. napus* were found to be differentially expressed compared to the water control following inoculation by the pathogens. This led to the identification of four plant DEG sets: (i) up-regulated genes in response to Lmb infection, (ii) down-regulated genes in response to Lmb infection, (iii) up-regulated genes in response to Lbb infection, and (iv) down-regulated genes in response to Lbb infection (Fig. 4a). By crossing these gene sets, we identified six sets of *B. napus* genes mobilized or repressed specifically in response to the Lmb or the Lbb infection (Fig. 4a,b).

A comparable number of genes were found to be specifically up-regulated in response to Lmb (2,362 genes) or Lbb SSI (1,946 genes) (Fig. 4a). In contrast, a much larger number of genes were down-regulated following infection by Lmb (5,351 genes) compared to Lbb SSI (1,843 genes) (Fig. 4a), reflecting manipulation of the plant immunity by Lmb.

The analysis of specific up- or down-regulated genes in *B. napus* was done here to detect if some responses activated or repressed by Lbb infection could negatively and indirectly impact the Lmb development. Thus, in the gene set specifically up-regulated in response to Lbb SSI + MSI, peptides, ribosomes or macromolecules biosynthesis processes (related the proteins production processes, *p* = 1.10^-100^; Additional file 10: S6 Fig) were over-represented, without clear evidence that these processes could impact colonization by Lmb. The main difference regarded the specific down regulation of numerous genes involved in responses to hormonal signalization following Lbb SSI +MSI (response to endogeneous stimuli, response to hormone, hormone mediated signaling pathways all *p* > 1.10^-20^ Additional file 12: S8 Fig).

Up-regulated genes specifically induced in response to Lmb were enriched in molecular transports (Golgi transport, protein transport, *p* = 4.10^-9^, Additional file 10: S6 Fig) and in global response to stress, temperature, and to chitin compounds (all *p <* 1.10^-8^, Additional file 10: S6 Fig). Specific enrichments were found only in response to Lmb inoculation, such as a set of 38 serine/threonine kinase proteins and genes belonging to the LOX pathway involved in the biosynthesis of Jasmonic Acid (*p* = 1.10^-2^; Additional file 11: S7 Fig). In the gene set specifically down-regulated in response to Lmb, genes involved in the nitrogen metabolism and RNA metabolism were over-represented (cellular nitrogen compound metabolic process; organonitrogen compound biosynthetic process; cellular nitrogen compound biosynthetic process all *p <* 1.10^-20^ Additional file 12: S8 Fig).
