# Supplementary material for: Location and timing govern tripartite interactions of fungal phytopathogens and host in the stem canker species complex
Source: BMC Biol. 2023 Nov 7;21:247. doi: 10.1186/s12915-023-01726-8 (PMC10631019; doi:10.1186/s12915-023-01726-8)
Supplement: Supplementary file 15 — Additional file 15: Fig. S10. Expression profiles of the 1,233 genes of Leptosphaeria biglobosa ‘brassicae’ (Lbb) detected as differentially expressed during the 15-days infection of Brassica napus cotyledons either following single species inoculation (SSI) or when inoculated as a mix (MSI) with Leptosphaeria maculans ‘brassicae’. Differentially expressed genes (DEGs) were detected using three different comparisons on samples obtained from the SSI inoculation of Lbb : 2 dpi vs 5-7-9-12-15 dpi (816 genes, black bar on the left of the heatmap), 5-7-9 dpi vs. 2-12-15 dpi (130 genes, orange bar on the left of the heatmap) and 12-15 dpi vs 2-5-7-9 dpi (286 genes, blue bar on the left of the heatmap). Gene raw counts of Lbb in SSI and MSI conditions were Log2(FPKM +1) transformed and then centered. The heatmap represents the normalized expression of DEGs detected on SSI and their expression are also shown during MSI. Genes annotated in the Small Secreted Protein repertoire are shown on the immediate left of the heatmap as black bars. Five categories of the “Molecular Function” Gene Ontology have a significant enrichment and are identified by colored bars on the left of the heatmap (Catalytic activities in black; Chitin binding in cyan; Glycosyl hydrolase activity in blue; Pectate lyase activity in red; Transmembrane transport activity in brown). [file 12915_2023_1726_MOESM15_ESM.pptx]

## Slide 1
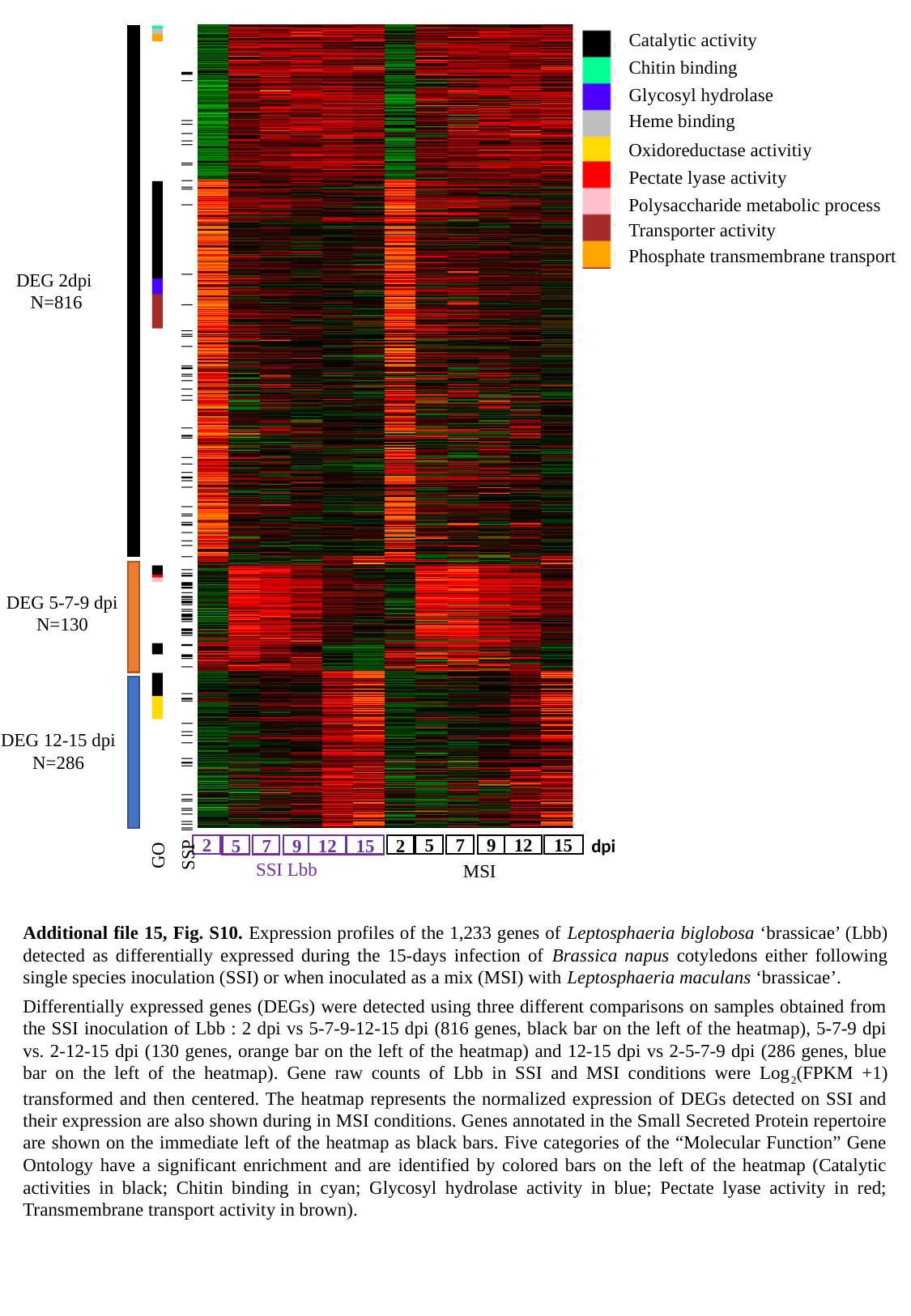

Catalytic activity
Chitin binding
Glycosyl hydrolase
Heme binding
Oxidoreductase activitiy
Pectate lyase activity
Polysaccharide metabolic process
Transporter activity
Phosphate transmembrane transport
DEG 2dpi
N=816
DEG 5-7-9 dpi
N=130
DEG 12-15 dpi
N=286
dpi
GO
SSP
2
5
7
9
12
15
5
7
9
12
15
2
SSI Lbb
MSI
Additional file 15, Fig. S10. Expression profiles of the 1,233 genes of Leptosphaeria biglobosa ‘brassicae’ (Lbb) detected as differentially expressed during the 15-days infection of Brassica napus cotyledons either following single species inoculation (SSI) or when inoculated as a mix (MSI) with Leptosphaeria maculans ‘brassicae’.
Differentially expressed genes (DEGs) were detected using three different comparisons on samples obtained from the SSI inoculation of Lbb : 2 dpi vs 5-7-9-12-15 dpi (816 genes, black bar on the left of the heatmap), 5-7-9 dpi vs. 2-12-15 dpi (130 genes, orange bar on the left of the heatmap) and 12-15 dpi vs 2-5-7-9 dpi (286 genes, blue bar on the left of the heatmap). Gene raw counts of Lbb in SSI and MSI conditions were Log2(FPKM +1) transformed and then centered. The heatmap represents the normalized expression of DEGs detected on SSI and their expression are also shown during in MSI conditions. Genes annotated in the Small Secreted Protein repertoire are shown on the immediate left of the heatmap as black bars. Five categories of the “Molecular Function” Gene Ontology have a significant enrichment and are identified by colored bars on the left of the heatmap (Catalytic activities in black; Chitin binding in cyan; Glycosyl hydrolase activity in blue; Pectate lyase activity in red; Transmembrane transport activity in brown).
