# Supplementary material for: Location and timing govern tripartite interactions of fungal phytopathogens and host in the stem canker species complex
Source: BMC Biol. 2023 Nov 7;21:247. doi: 10.1186/s12915-023-01726-8 (PMC10631019; doi:10.1186/s12915-023-01726-8)
Supplement: Supplementary file 16 — Additional file 16: Fig. S11. Detection of Gene Ontology enrichments (“Molecular Function” category) among Leptosphaeria biglobosa ‘brassicae’ down-regulated gene set (green) or up-regulated gene set (red) during infection of cotyledons of Brassica napus. For each of the three stages depicted in Fig. 3 (2 days post-inoculation (dpi), 5-7-9 dpi, and 12-15 dpi), GO enrichment in the set of genes up-regulated (red) or down-regulated (green) was identified using a hypergeometrical test with the Cytoscape tool Bingo. The y axis indicates the terms overrepresented in the Molecular function category. The x axis represents the -Log10(FDR) of the enrichment test. The numbers in the boxes indicate the number of genes assigned to the corresponding Molecular Function in the cluster (left) and the total number of genes associated to this Molecular Function term in the whole genome gene set (right). [file 12915_2023_1726_MOESM16_ESM.pptx]

## Slide 1
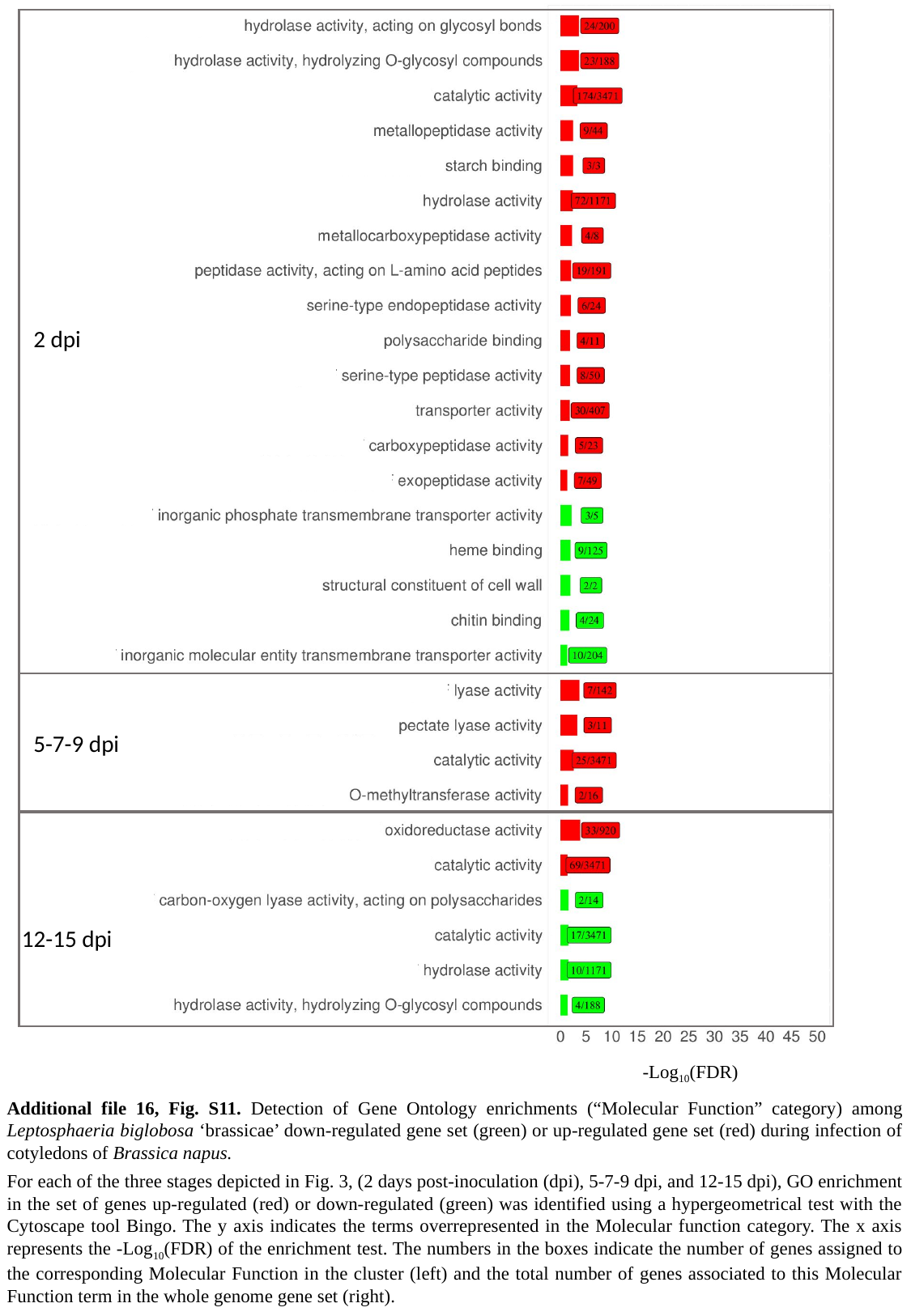

2 dpi
5-7-9 dpi
12-15 dpi
-Log10(FDR)
Additional file 16, Fig. S11. Detection of Gene Ontology enrichments (“Molecular Function” category) among Leptosphaeria biglobosa ‘brassicae’ down-regulated gene set (green) or up-regulated gene set (red) during infection of cotyledons of Brassica napus.
For each of the three stages depicted in Fig. 3, (2 days post-inoculation (dpi), 5-7-9 dpi, and 12-15 dpi), GO enrichment in the set of genes up-regulated (red) or down-regulated (green) was identified using a hypergeometrical test with the Cytoscape tool Bingo. The y axis indicates the terms overrepresented in the Molecular function category. The x axis represents the -Log10(FDR) of the enrichment test. The numbers in the boxes indicate the number of genes assigned to the corresponding Molecular Function in the cluster (left) and the total number of genes associated to this Molecular Function term in the whole genome gene set (right).
