# Supplementary material for: Location and timing govern tripartite interactions of fungal phytopathogens and host in the stem canker species complex
Source: BMC Biol. 2023 Nov 7;21:247. doi: 10.1186/s12915-023-01726-8 (PMC10631019; doi:10.1186/s12915-023-01726-8)
Supplement: Supplementary file 18 — Additional file 18: Table S4. Enrichment analyses of the proportion of genes encoding Small Secreted Proteins (SSP) in the Differentially expressed gene (DEG) sets detected in Leptosphaeria biglobosa ‘brassicae’ during cotyledon infections. a To detect if genes encoding SSPs were over-represented in DEGs, their proportion was compared to those of SSPs in the entire gene set of Lbb by a Chi-Squared test (** : p-value < 0.001). b To detect if a specific enrichment of genes encoding SSPs was found in each group of DEGs (2 dpi; 5-9 dpi; 12-15 dpi), their proportion was compared to the proportion of SSPs in the total DEGs set by a Chi-Squared test (** : p-value < 0.001, *: p-value < 0.05). [file 12915_2023_1726_MOESM18_ESM.pptx]

## Slide 1
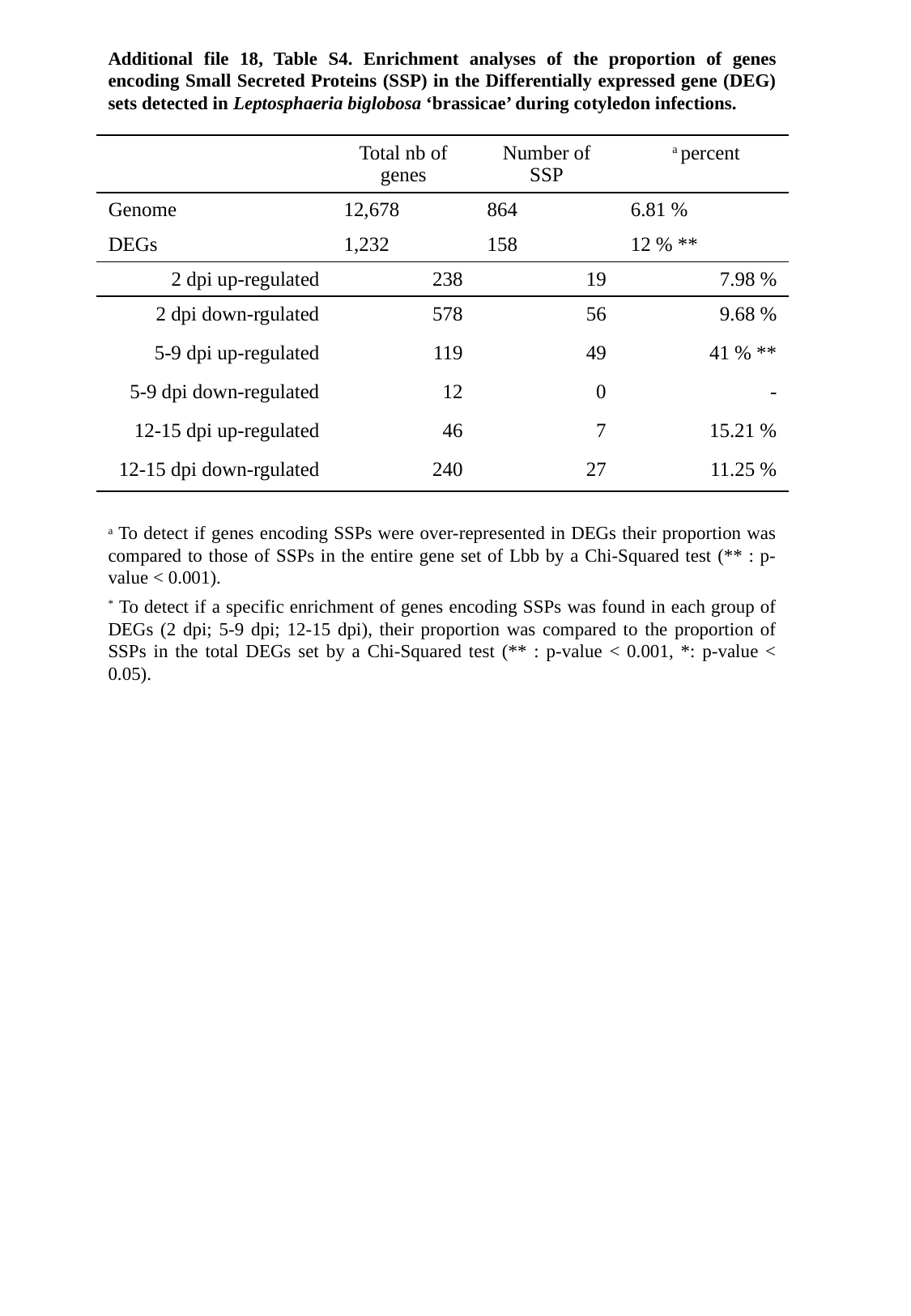

Additional file 18, Table S4. Enrichment analyses of the proportion of genes encoding Small Secreted Proteins (SSP) in the Differentially expressed gene (DEG) sets detected in Leptosphaeria biglobosa ‘brassicae’ during cotyledon infections.
| | Total nb of genes | Number of SSP | a percent |
| --- | --- | --- | --- |
| Genome | 12,678 | 864 | 6.81 % |
| DEGs | 1,232 | 158 | 12 % \*\* |
| 2 dpi up-regulated | 238 | 19 | 7.98 % |
| 2 dpi down-rgulated | 578 | 56 | 9.68 % |
| 5-9 dpi up-regulated | 119 | 49 | 41 % \*\* |
| 5-9 dpi down-regulated | 12 | 0 | - |
| 12-15 dpi up-regulated | 46 | 7 | 15.21 % |
| 12-15 dpi down-rgulated | 240 | 27 | 11.25 % |
a To detect if genes encoding SSPs were over-represented in DEGs their proportion was compared to those of SSPs in the entire gene set of Lbb by a Chi-Squared test (** : p-value < 0.001).
* To detect if a specific enrichment of genes encoding SSPs was found in each group of DEGs (2 dpi; 5-9 dpi; 12-15 dpi), their proportion was compared to the proportion of SSPs in the total DEGs set by a Chi-Squared test (** : p-value < 0.001, *: p-value < 0.05).
