# Supplementary material for: Location and timing govern tripartite interactions of fungal phytopathogens and host in the stem canker species complex
Source: BMC Biol. 2023 Nov 7;21:247. doi: 10.1186/s12915-023-01726-8 (PMC10631019; doi:10.1186/s12915-023-01726-8)
Supplement: Supplementary file 19 — Additional file 19: Fig. S13. Detection of Gene Ontology enrichments (“Molecular Function” category) among Leptosphaeria maculans ‘brassicae’ (Lmb) down-regulated gene set (green) or up-regulated gene set (red) during Mixed Species Inoculation (MSI) compared to Single Species Inoculation (SSI). For each time point of the kinetics of infection (5, 7, 9, 12 or 15 days post-inoculation-dpi), GO enrichment in the set of genes up-regulated (red) or down-regulated (green) in Lmb during MSI compared to SSI was identified using a hypergeometrical test with the Cytoscape tool Bingo. The y axis indicates the terms overrepresented in the Molecular Function category. The x axis represents the -Log10(FDR) of the enrichment test. The numbers in the boxes indicate the number of genes assigned to the corresponding Molecular Function in the cluster (left) and the total number of genes associated to this Biological Process term in the whole genome gene set (right). [file 12915_2023_1726_MOESM19_ESM.pptx]

## Slide 1
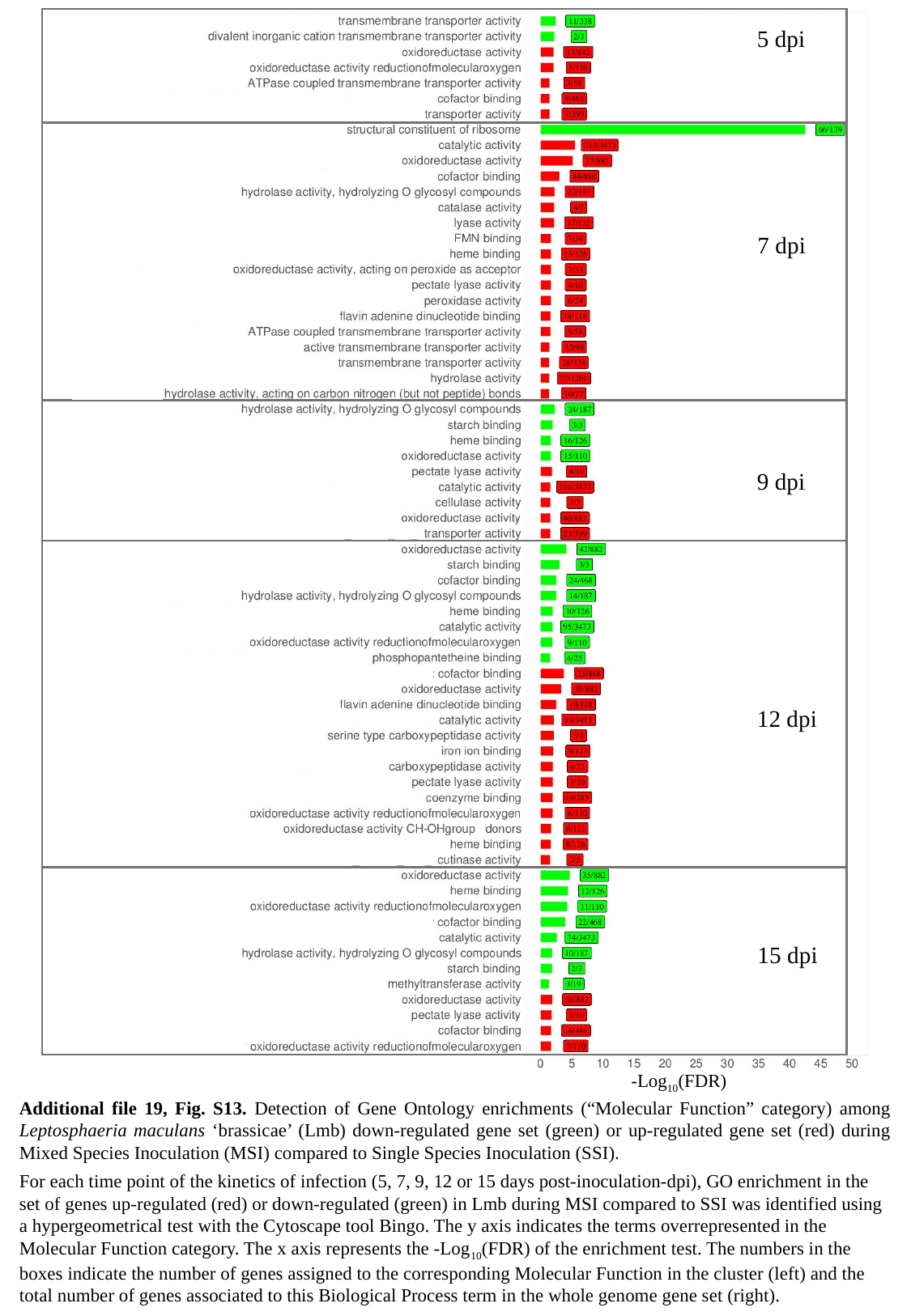

5 dpi
7 dpi
9 dpi
12 dpi
15 dpi
-Log10(FDR)
Additional file 19, Fig. S13. Detection of Gene Ontology enrichments (“Molecular Function” category) among Leptosphaeria maculans ‘brassicae’ (Lmb) down-regulated gene set (green) or up-regulated gene set (red) during Mixed Species Inoculation (MSI) compared to Single Species Inoculation (SSI).
For each time point of the kinetics of infection (5, 7, 9, 12 or 15 days post-inoculation-dpi), GO enrichment in the set of genes up-regulated (red) or down-regulated (green) in Lmb during MSI compared to SSI was identified using a hypergeometrical test with the Cytoscape tool Bingo. The y axis indicates the terms overrepresented in the Molecular Function category. The x axis represents the -Log10(FDR) of the enrichment test. The numbers in the boxes indicate the number of genes assigned to the corresponding Molecular Function in the cluster (left) and the total number of genes associated to this Biological Process term in the whole genome gene set (right).
