# Supplementary material for: Location and timing govern tripartite interactions of fungal phytopathogens and host in the stem canker species complex
Source: BMC Biol. 2023 Nov 7;21:247. doi: 10.1186/s12915-023-01726-8 (PMC10631019; doi:10.1186/s12915-023-01726-8)
Supplement: Supplementary file 22 — Additional file 22: Table S5. Modification of Leptosphaeria maculans ‘brassicae’ (Lmb) gene expression during plant infection in the presence of Leptosphaeria biglobosa ‘brassicae’ (Lbb; Mixed Species Inoculation : MSI). a Total number of genes in the Lmb genome and total number of genes specifically expressed during the infectious cycle [28]. b The proportion of Differentially expressed genes (DEGs) in MSI (17.7% of the total gene set) was compared to the proportion of DEGs in MSI in the 1,203 gene set (51.8%) by Chi-Squared test (**: p < 0.001, *: p < 0.05). c As described by Gay et al. [28], the 1207 genes specifically expressed by Lmb during plant infection are grouped into eight expression waves representative of the fungal lifestyle and/or of specific organs colonized. d To detect which expression waves was significantly impacted by the MSI conditions, the proportion of DEGs in each wave was compared to the total proportion of DEG in the 1,207 genes involved in the infectious cycle using a Chi-Squared test (**: p < 0.001, *: p < 0.05). Three waves (1, 2 and 3) were highly enriched in DEGs during MSI condition (in bold). Two clusters (5 and 6 corresponding to stem infection) were underrepresented in the DEGs in MSI conditions. e To detect if genes in clusters 1, 2 and 3 trended to be up- or downregulated in MSI conditions, a second Chi-Squared test (**: p < 0.001, *: p < 0.05) was done by comparing the proportion of down- and upregulated genes in each cluster in MSI to the total amount of down- and upregulated genes among the 1,207 genes set. f Nd : not done, statistical tests were only performed on overrepresented clusters. [file 12915_2023_1726_MOESM22_ESM.pptx]

## Slide 1
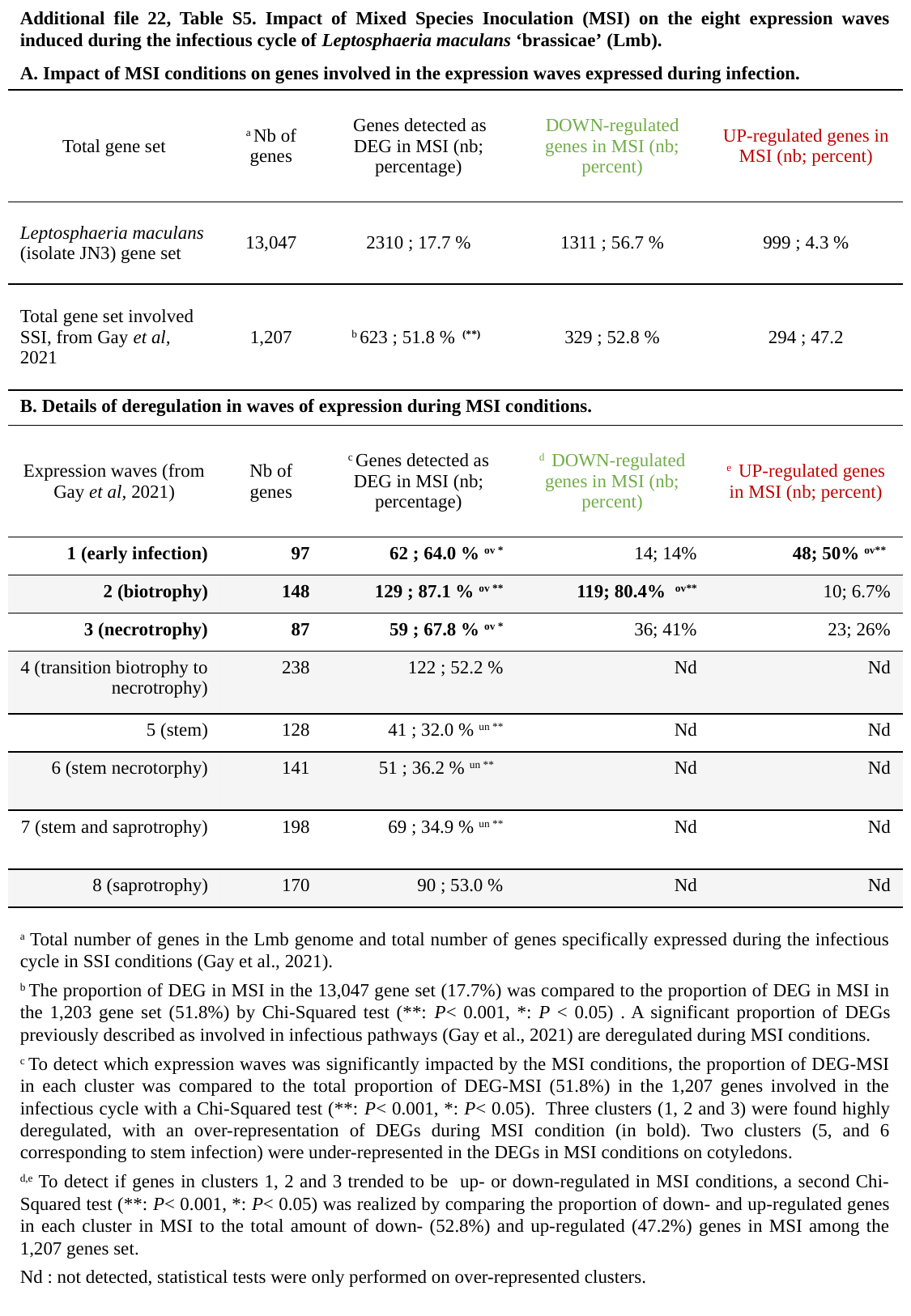

Additional file 22, Table S5. Impact of Mixed Species Inoculation (MSI) on the eight expression waves induced during the infectious cycle of Leptosphaeria maculans ‘brassicae’ (Lmb).
| A. Impact of MSI conditions on genes involved in the expression waves expressed during infection. | | | | |
| --- | --- | --- | --- | --- |
| Total gene set | a Nb of genes | Genes detected as DEG in MSI (nb; percentage) | DOWN-regulated genes in MSI (nb; percent) | UP-regulated genes in MSI (nb; percent) |
| Leptosphaeria maculans (isolate JN3) gene set | 13,047 | 2310 ; 17.7 % | 1311 ; 56.7 % | 999 ; 4.3 % |
| Total gene set involved SSI, from Gay et al, 2021 | 1,207 | b 623 ; 51.8 % (\*\*) | 329 ; 52.8 % | 294 ; 47.2 |
| B. Details of deregulation in waves of expression during MSI conditions. | | | | |
| Expression waves (from Gay et al, 2021) | Nb of genes | c Genes detected as DEG in MSI (nb; percentage) | d DOWN-regulated genes in MSI (nb; percent) | e UP-regulated genes in MSI (nb; percent) |
| 1 (early infection) | 97 | 62 ; 64.0 % ov \* | 14; 14% | 48; 50% ov\*\* |
| 2 (biotrophy) | 148 | 129 ; 87.1 % ov \*\* | 119; 80.4% ov\*\* | 10; 6.7% |
| 3 (necrotrophy) | 87 | 59 ; 67.8 % ov \* | 36; 41% | 23; 26% |
| 4 (transition biotrophy to necrotrophy) | 238 | 122 ; 52.2 % | Nd | Nd |
| 5 (stem) | 128 | 41 ; 32.0 % un \*\* | Nd | Nd |
| 6 (stem necrotorphy) | 141 | 51 ; 36.2 % un \*\* | Nd | Nd |
| 7 (stem and saprotrophy) | 198 | 69 ; 34.9 % un \*\* | Nd | Nd |
| 8 (saprotrophy) | 170 | 90 ; 53.0 % | Nd | Nd |
a Total number of genes in the Lmb genome and total number of genes specifically expressed during the infectious cycle in SSI conditions (Gay et al., 2021).
b The proportion of DEG in MSI in the 13,047 gene set (17.7%) was compared to the proportion of DEG in MSI in the 1,203 gene set (51.8%) by Chi-Squared test (**: P< 0.001, *: P < 0.05) . A significant proportion of DEGs previously described as involved in infectious pathways (Gay et al., 2021) are deregulated during MSI conditions.
c To detect which expression waves was significantly impacted by the MSI conditions, the proportion of DEG-MSI in each cluster was compared to the total proportion of DEG-MSI (51.8%) in the 1,207 genes involved in the infectious cycle with a Chi-Squared test (**: P< 0.001, *: P< 0.05). Three clusters (1, 2 and 3) were found highly deregulated, with an over-representation of DEGs during MSI condition (in bold). Two clusters (5, and 6 corresponding to stem infection) were under-represented in the DEGs in MSI conditions on cotyledons.
d,e To detect if genes in clusters 1, 2 and 3 trended to be up- or down-regulated in MSI conditions, a second Chi-Squared test (**: P< 0.001, *: P< 0.05) was realized by comparing the proportion of down- and up-regulated genes in each cluster in MSI to the total amount of down- (52.8%) and up-regulated (47.2%) genes in MSI among the 1,207 genes set.
Nd : not detected, statistical tests were only performed on over-represented clusters.
